# Supplementary material for: In-silico prediction and modeling of the Entamoeba histolytica proteins: Serine-rich Entamoeba histolytica protein and 29 kDa Cysteine-rich protease
Source: PeerJ. 2017 Jun 28;5:e3160. doi: 10.7717/peerj.3160 (PMC5493030; doi:10.7717/peerj.3160)
Supplement: Supplemental Information 1 — The Ramachandran plot shows the percentage of amino acids in the allowed and disallowed regions for determining if the predicted structure is stable in nature. [file peerj-05-3160-s001.pdf]

PROCHECK

Ramachandran Plot

23_128_72

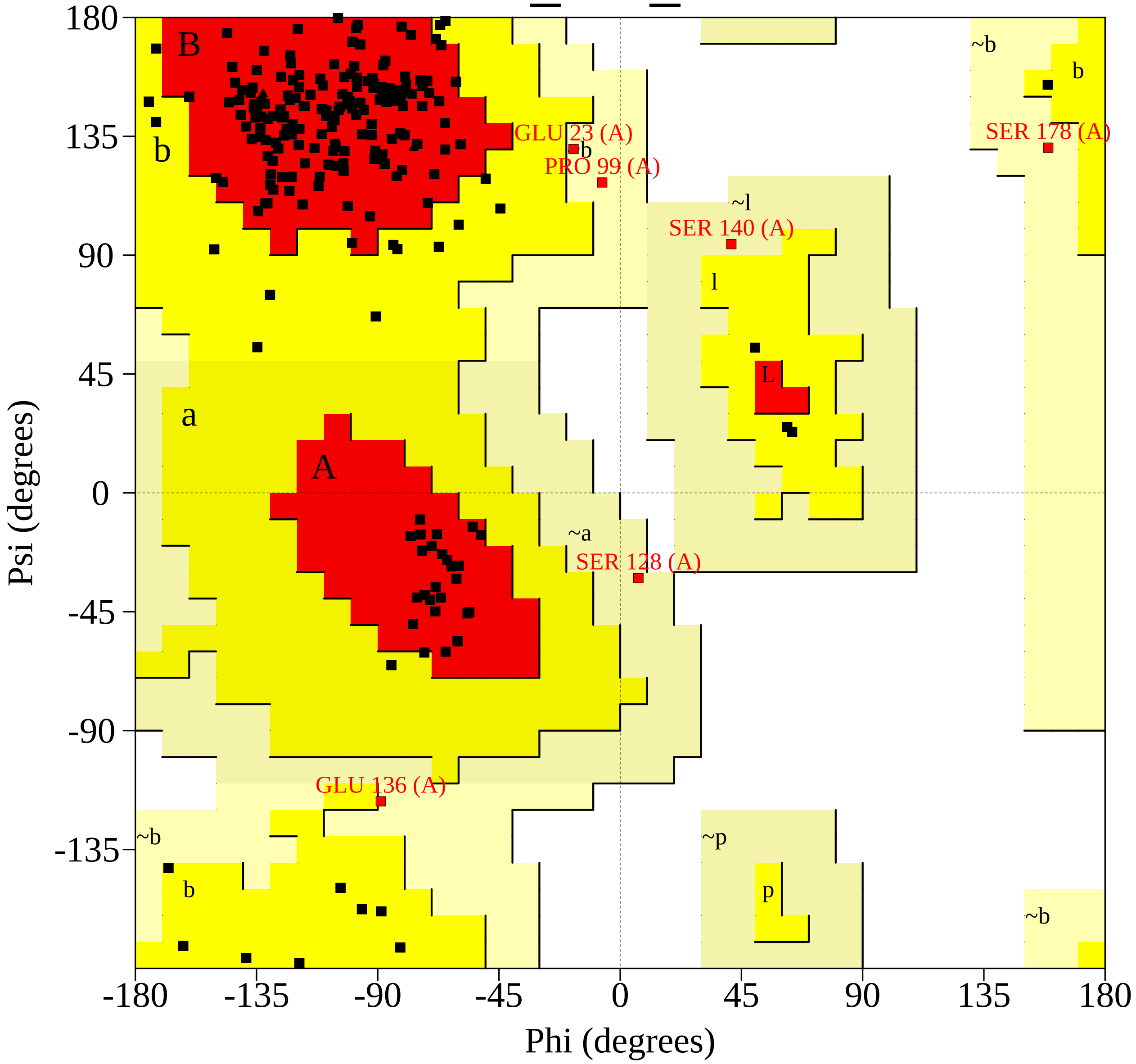

Plot statistics

Residues in most favoured regions [A,B,L]	177	84.7%
Residues in additional allowed regions [a,b,l,p]	27	12.9%
Residues in generously allowed regions [~a,~b,~l,~p]	5	2.4%
Residues in disallowed regions	0	0.0%
	----	------
Number of non-glycine and non-proline residues	209	100.0%
Number of end-residues (excl. Gly and Pro)	2	
Number of glycine residues (shown as triangles)	2	
Number of proline residues	20	

Total number of residues	233	

Based on an analysis of 118 structures of resolution of at least 2.0 Angstroms and R-factor no greater than 20%, a good quality model would be expected to have over 90% in the most favoured regions.

PROCHECK

Ramachandran Plot

Model_12

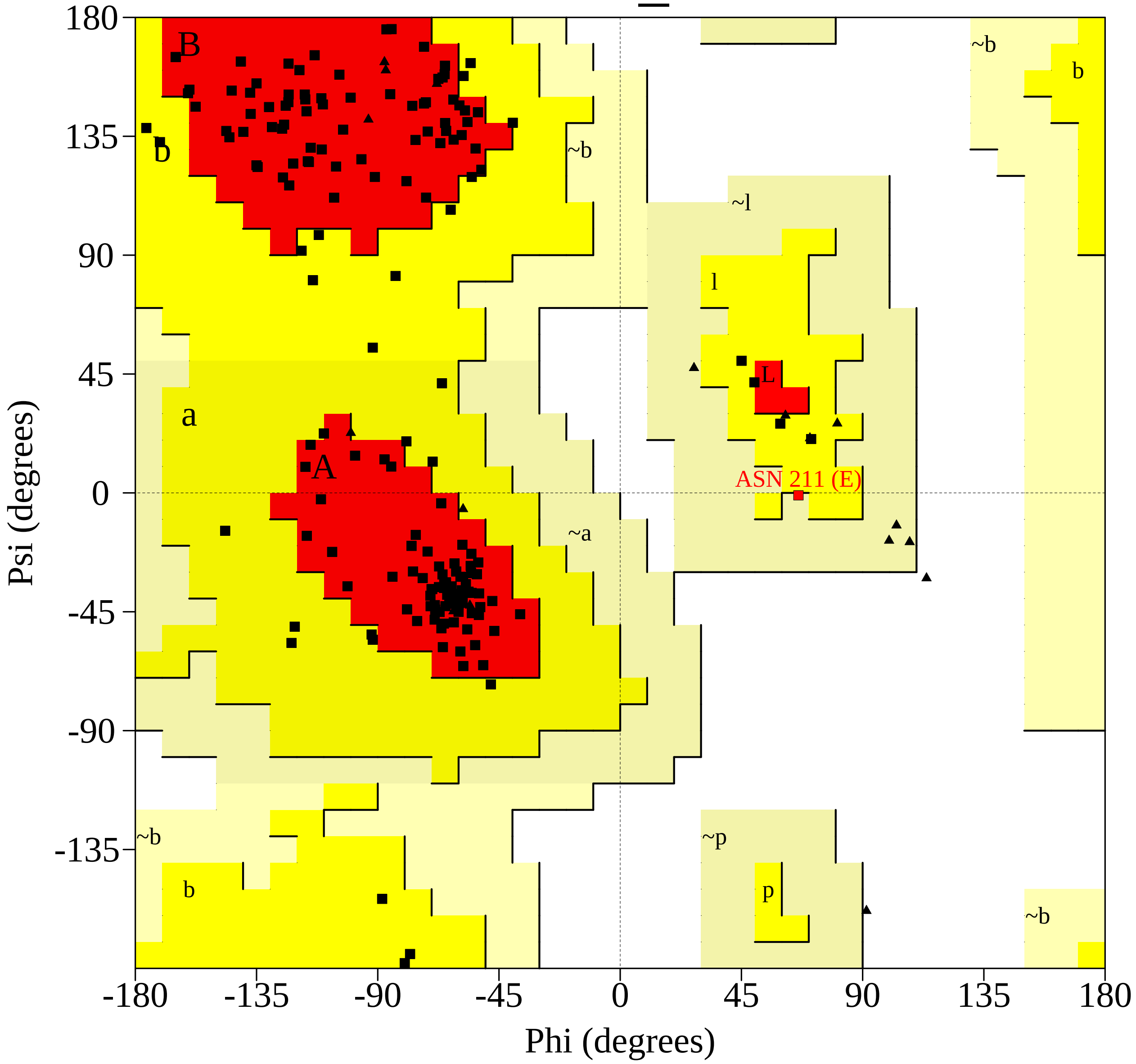

Plot statistics

Residues in most favoured regions [A,B,L]	139	84.8%
Residues in additional allowed regions [a,b,l,p]	24	14.6%
Residues in generously allowed regions [~a,~b,~l,~p]	1	0.6%
Residues in disallowed regions	0	0.0%
	----	------
Number of non-glycine and non-proline residues	164	100.0%
Number of end-residues (excl. Gly and Pro)	2	
Number of glycine residues (shown as triangles)	18	
Number of proline residues	10	

Total number of residues	194	

Based on an analysis of 118 structures of resolution of at least 2.0 Angstroms and R-factor no greater than 20%, a good quality model would be expected to have over 90% in the most favoured regions.

PROCHECK

Ramachandran plots for all residue types

23_128_72

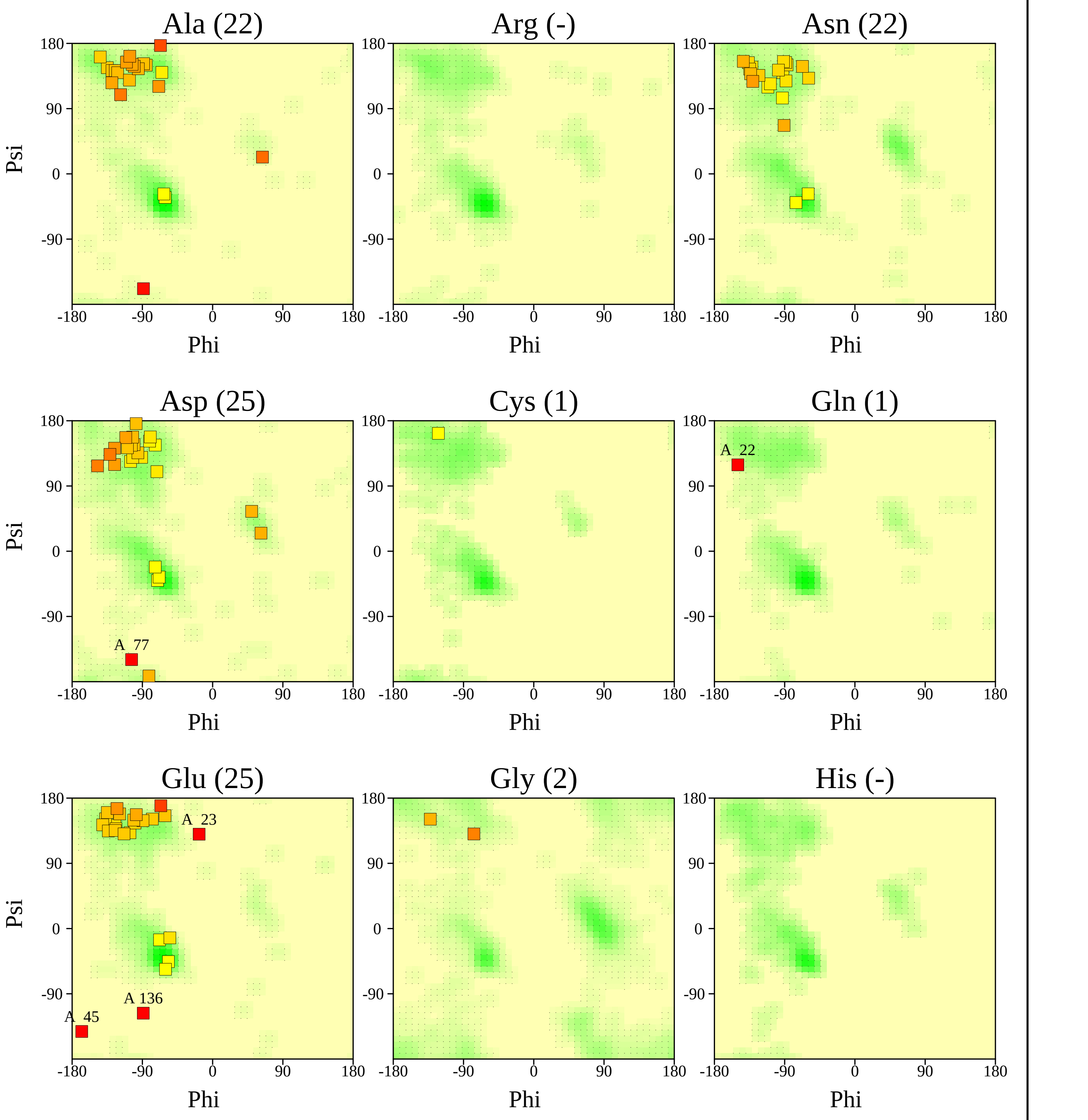

Numbers of residues are shown in brackets. Those in unfavourable conformations (score < -3.00) are labelled.
Shading shows favourable conformations as obtained from an analysis of 163 structures at resolution 2.0A or better.

Ramachandran plots for all residue types

23_128_72

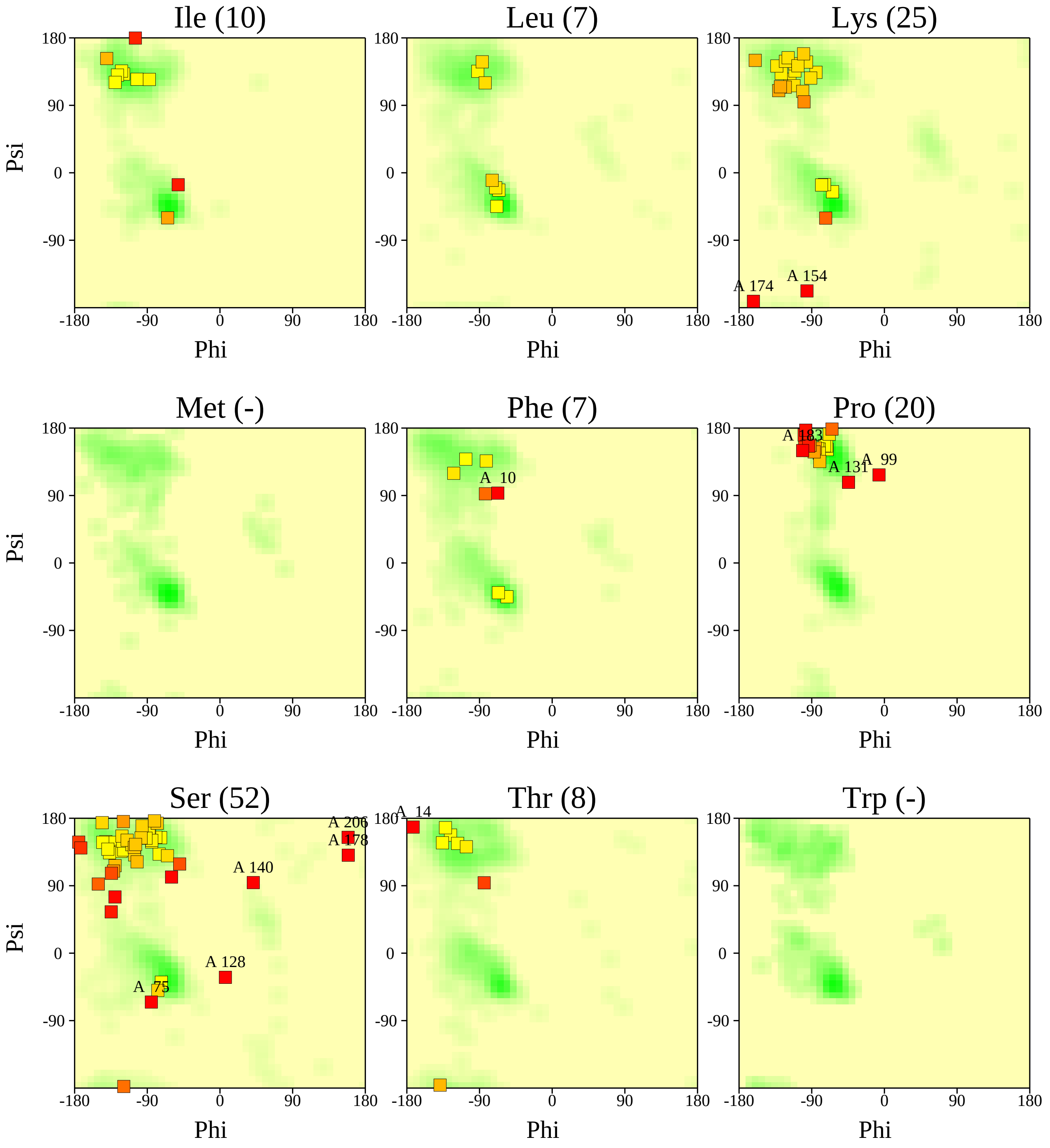

Numbers of residues are shown in brackets. Those in unfavourable conformations (score < -3.00) are labelled.
Shading shows favourable conformations as obtained from an analysis of 163 structures at resolution 2.0A or better.

Ramachandran plots for all residue types

23_128_72

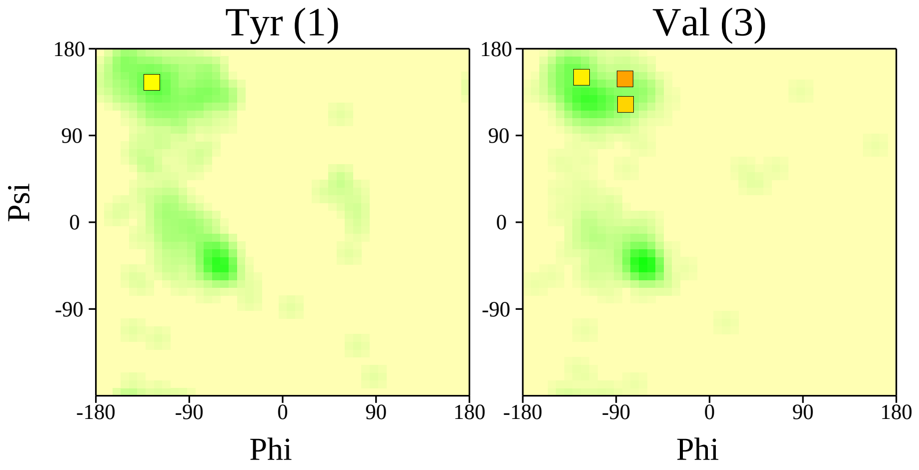

Numbers of residues are shown in brackets. Those in unfavourable conformations (score < -3.00) are labelled.
Shading shows favourable conformations as obtained from an analysis of 163 structures at resolution 2.0A or better.

Ramachandran plots for all residue types

Model_12

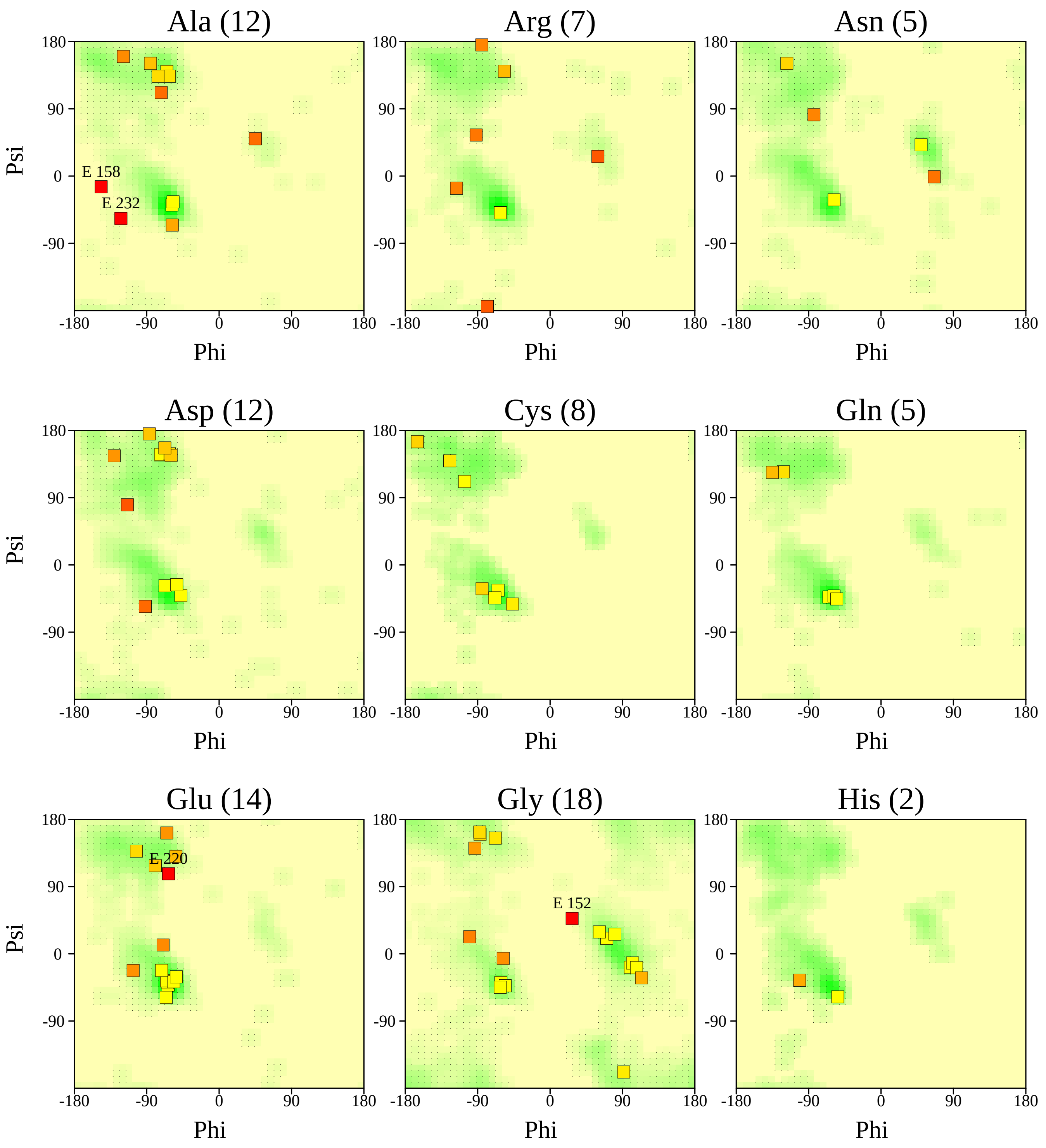

Numbers of residues are shown in brackets. Those in unfavourable conformations (score < -3.00) are labelled.
Shading shows favourable conformations as obtained from an analysis of 163 structures at resolution 2.0A or better.

Ramachandran plots for all residue types

Model_12

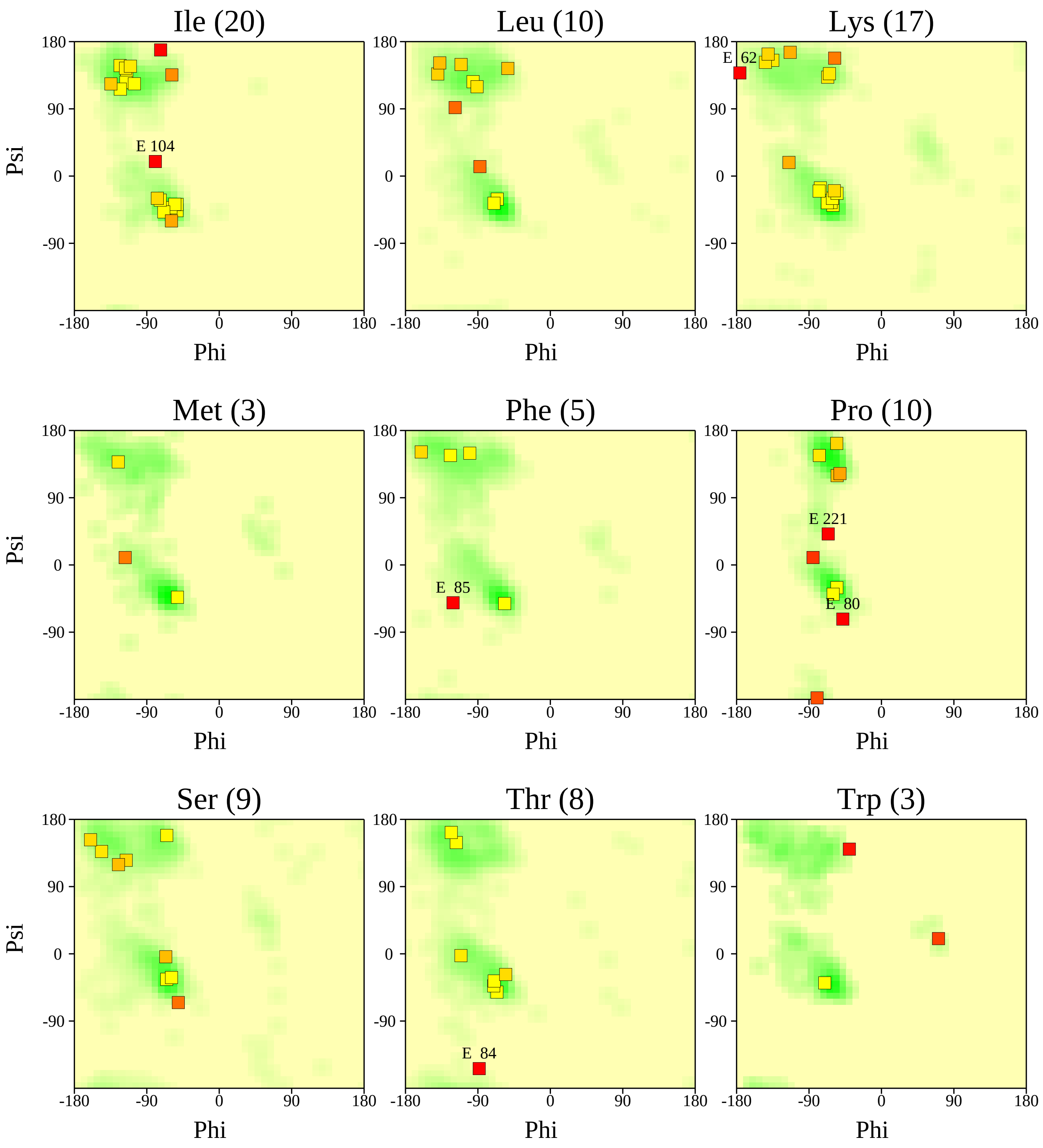

Numbers of residues are shown in brackets. Those in unfavourable conformations (score < -3.00) are labelled.
Shading shows favourable conformations as obtained from an analysis of 163 structures at resolution 2.0A or better.

Ramachandran plots for all residue types

Model_12

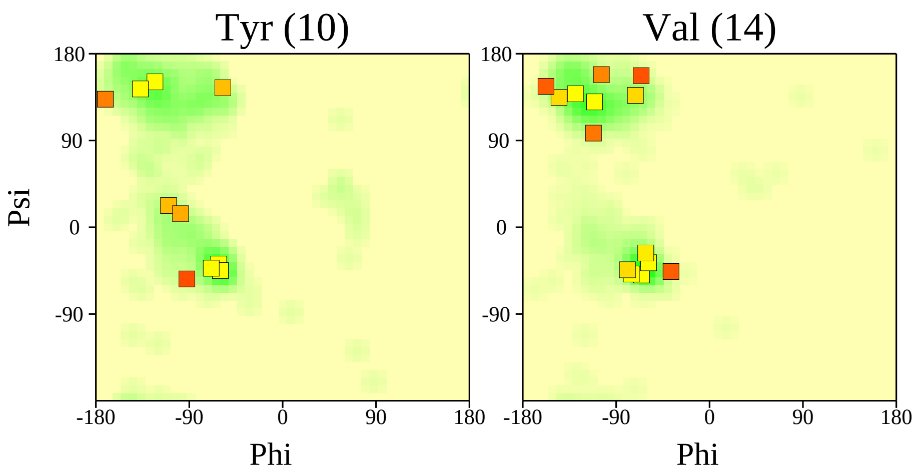

Numbers of residues are shown in brackets. Those in unfavourable conformations (score < -3.00) are labelled.
Shading shows favourable conformations as obtained from an analysis of 163 structures at resolution 2.0A or better.
