# Supplementary material for: In-silico prediction and modeling of the Entamoeba histolytica proteins: Serine-rich Entamoeba histolytica protein and 29 kDa Cysteine-rich protease
Source: PeerJ. 2017 Jun 28;5:e3160. doi: 10.7717/peerj.3160 (PMC5493030; doi:10.7717/peerj.3160)

PROVE

23_128_72.pdb

Analysis of entire structure

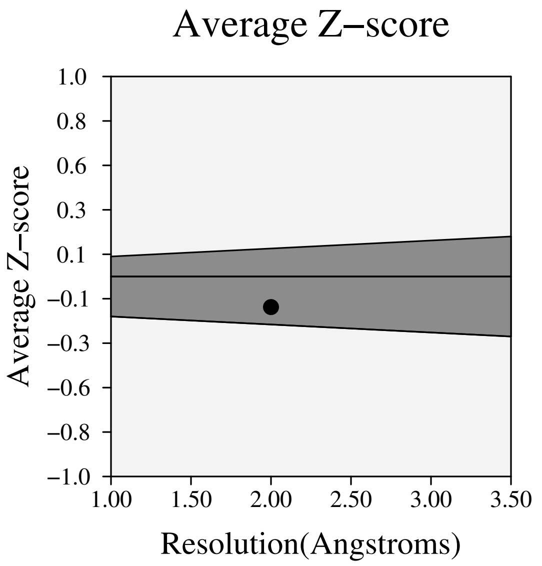

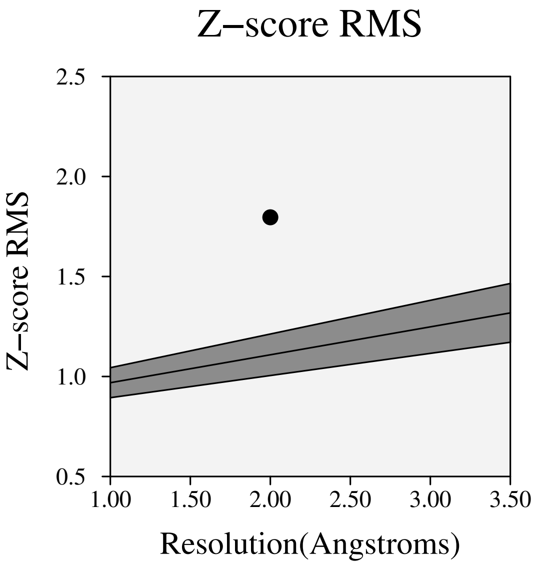

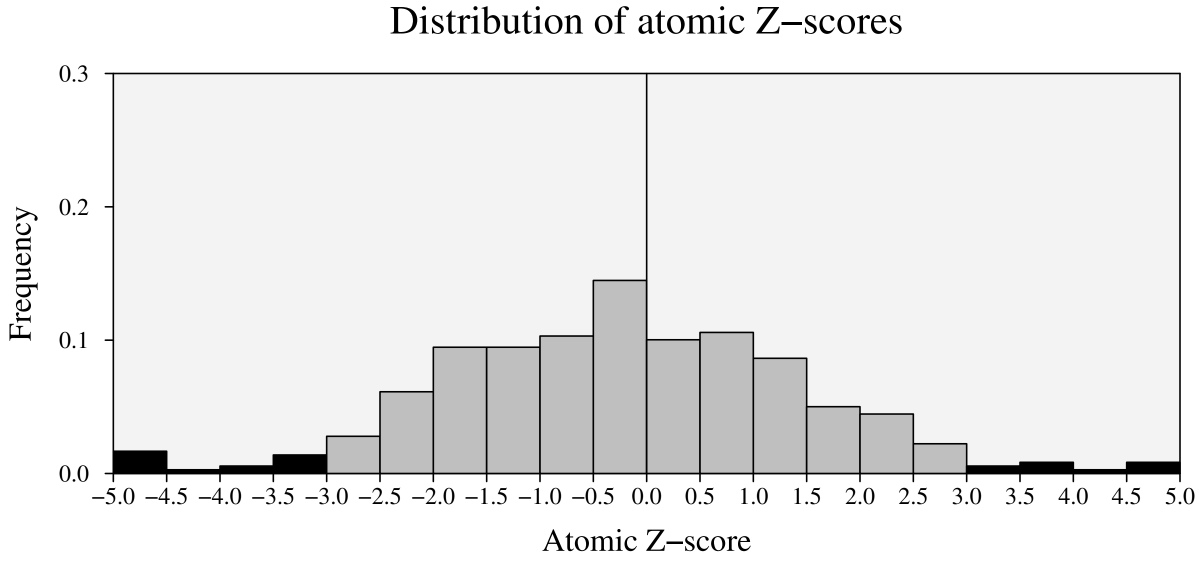

Z−score mean	−0.153
Z−score stddev	1.791
Z−score RMS	1.796
# scored atoms	438
# outliers	33
% outliers	7.500

Pontius et al. (1996) J. Mol. Biol. 264, 121−136

PROVE

23_128_72.pdb

Analysis of residues

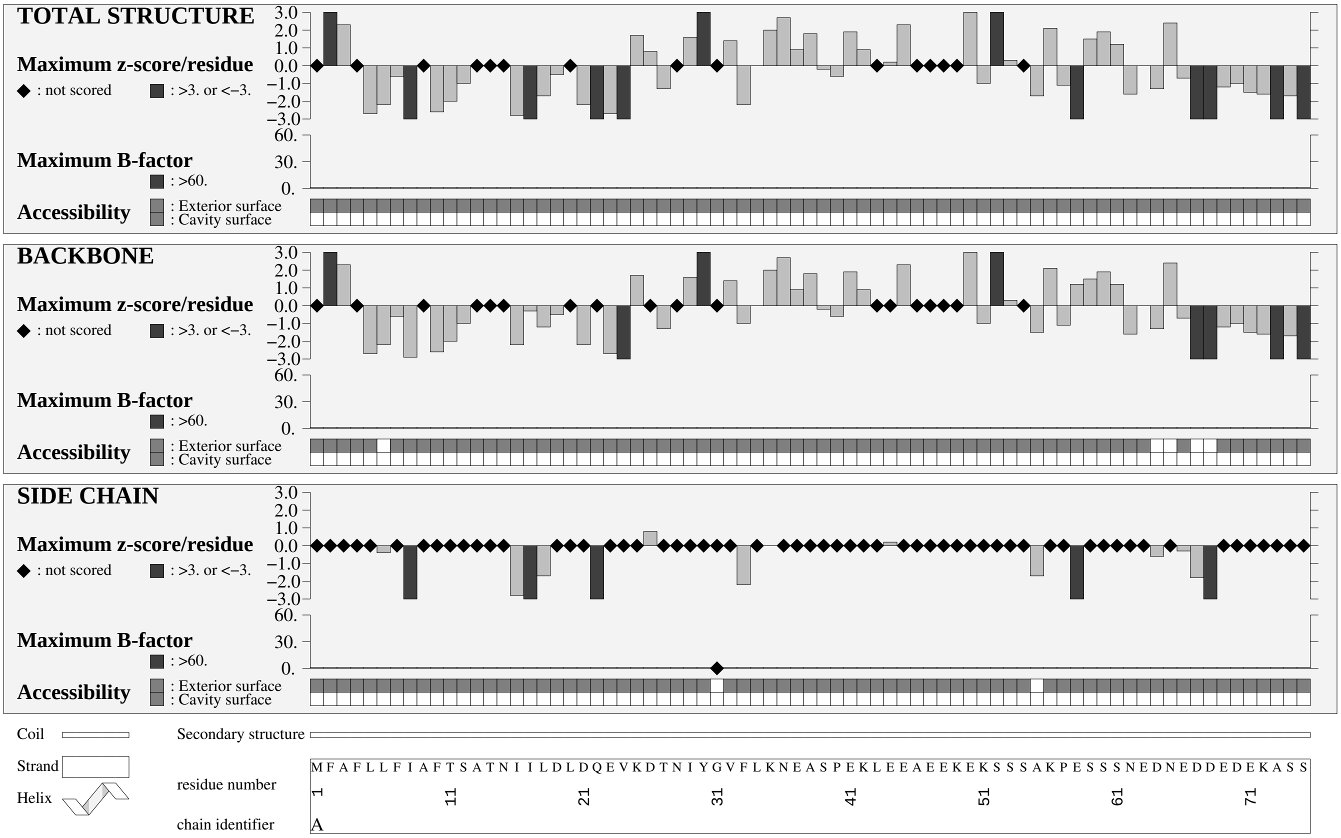

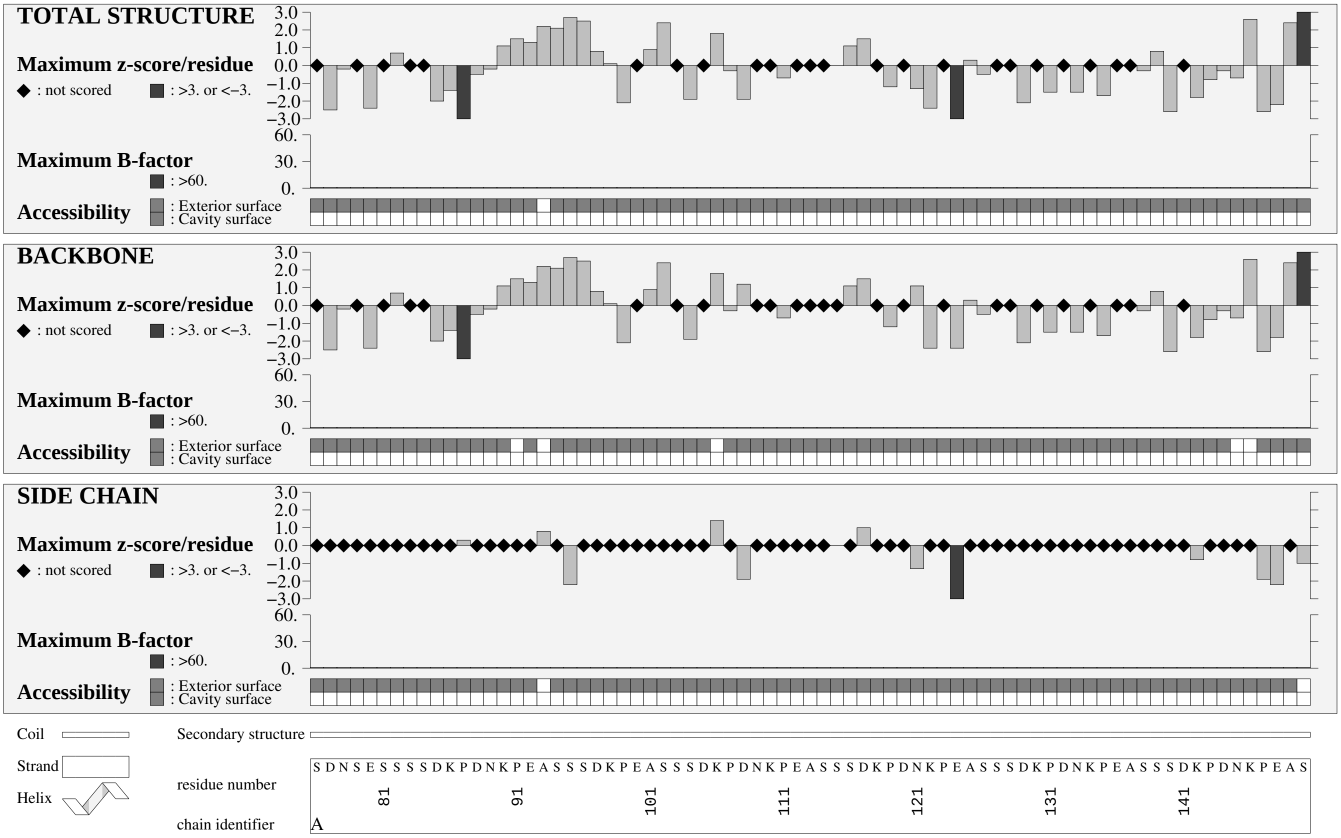

PROVE

23_128_72.pdb

Analysis of residues (2)

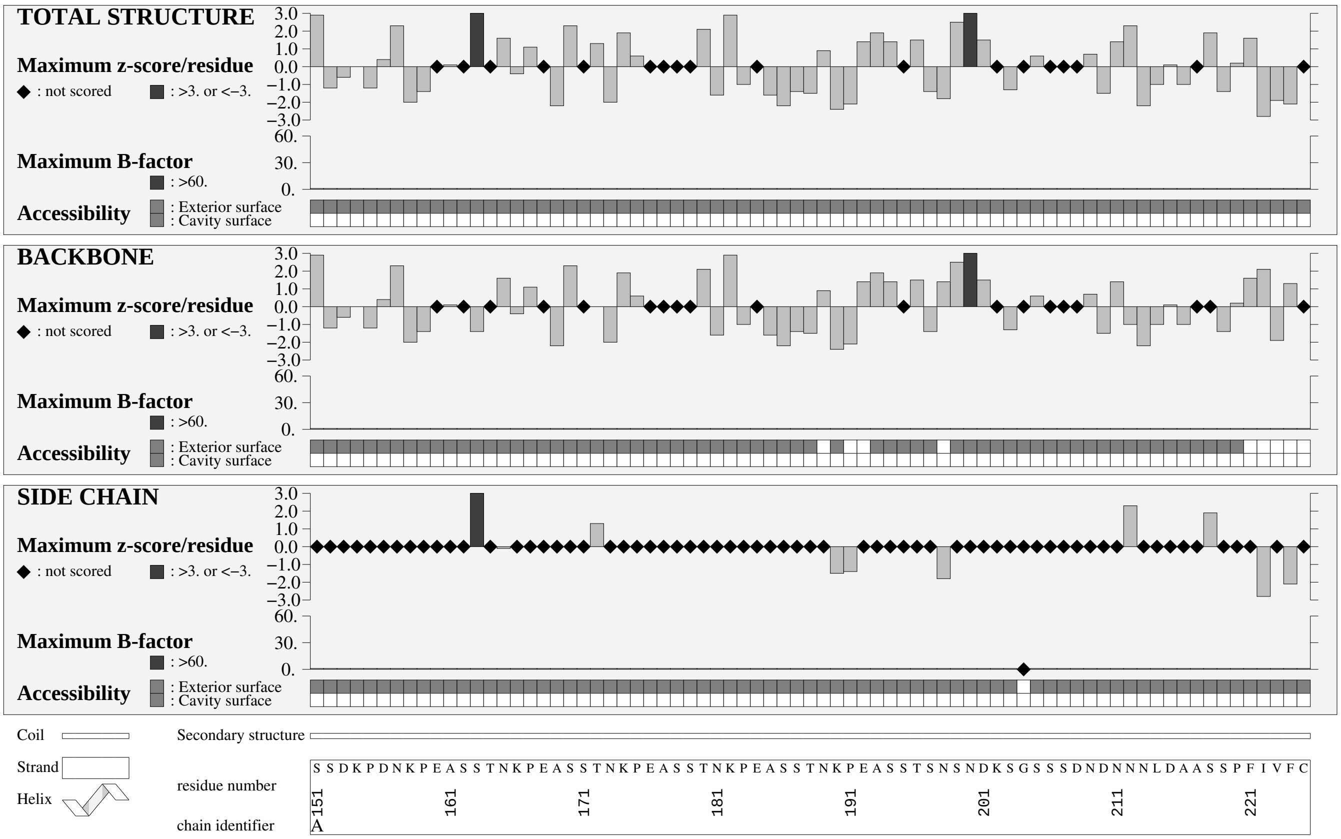

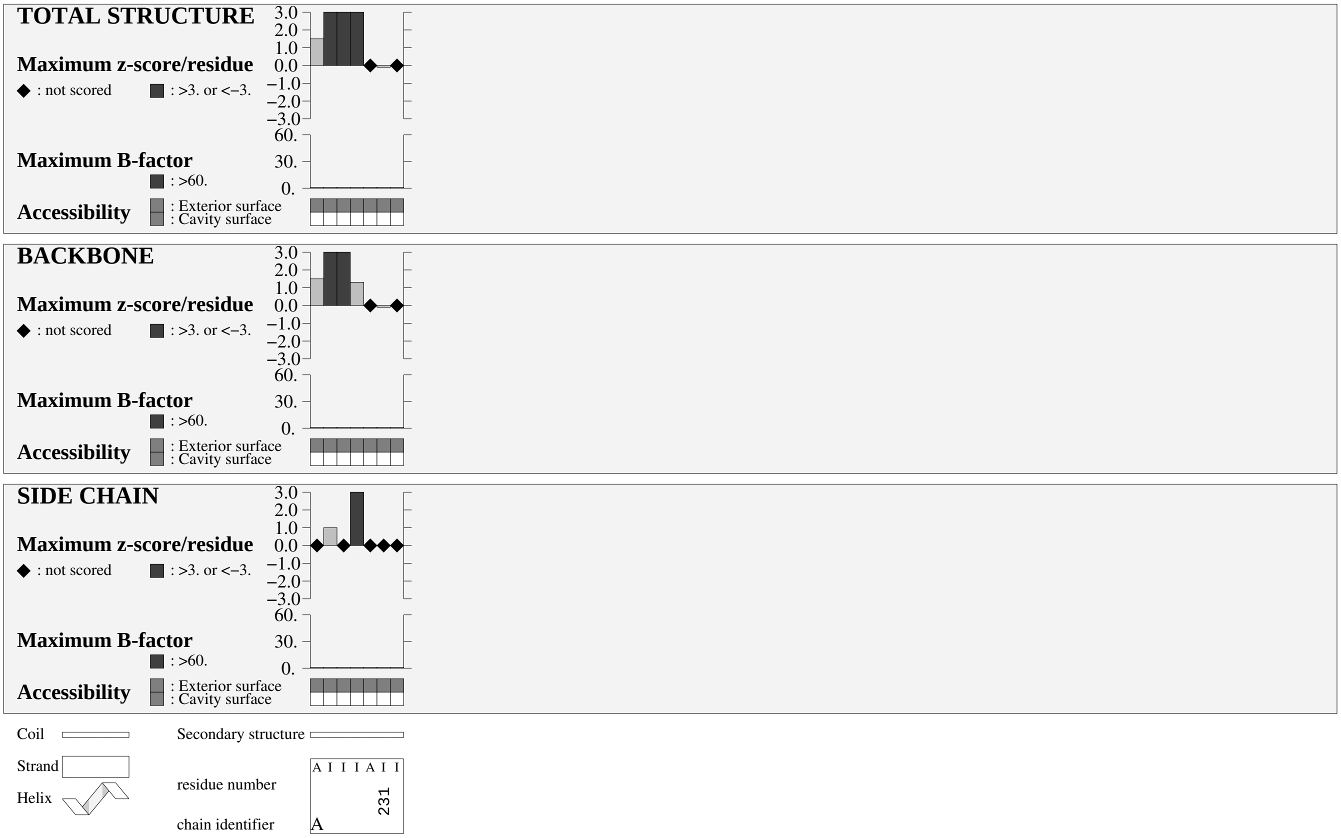

PROVE

Model_12.pdb

Analysis of entire structure

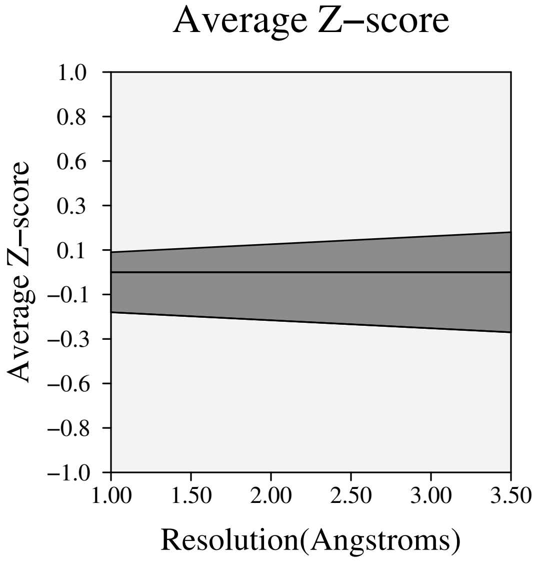

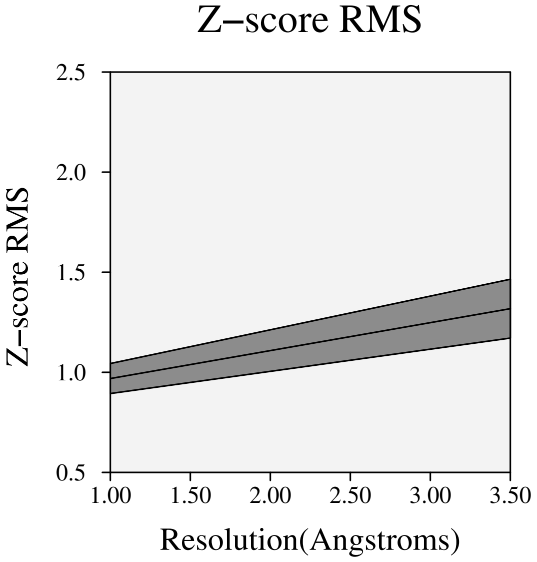

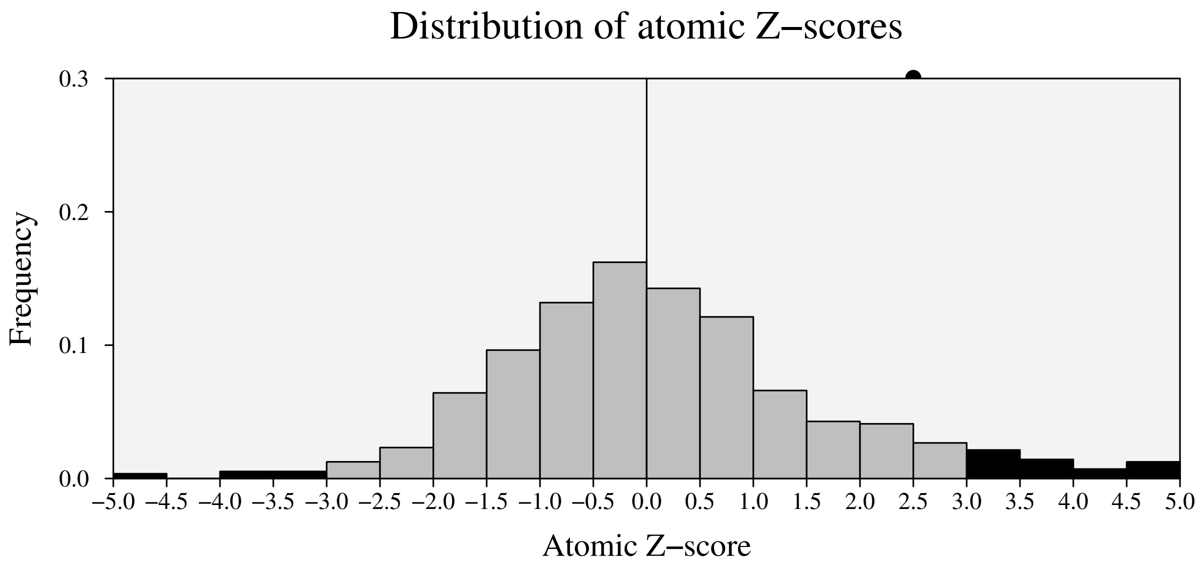

Z−score mean	6.446
Z−score stddev	79.103
Z−score RMS	79.303
# scored atoms	635
# outliers	42
% outliers	6.600

Pontius et al. (1996) J. Mol. Biol. 264, 121−136

PROVE

Model_12.pdb

Analysis of residues

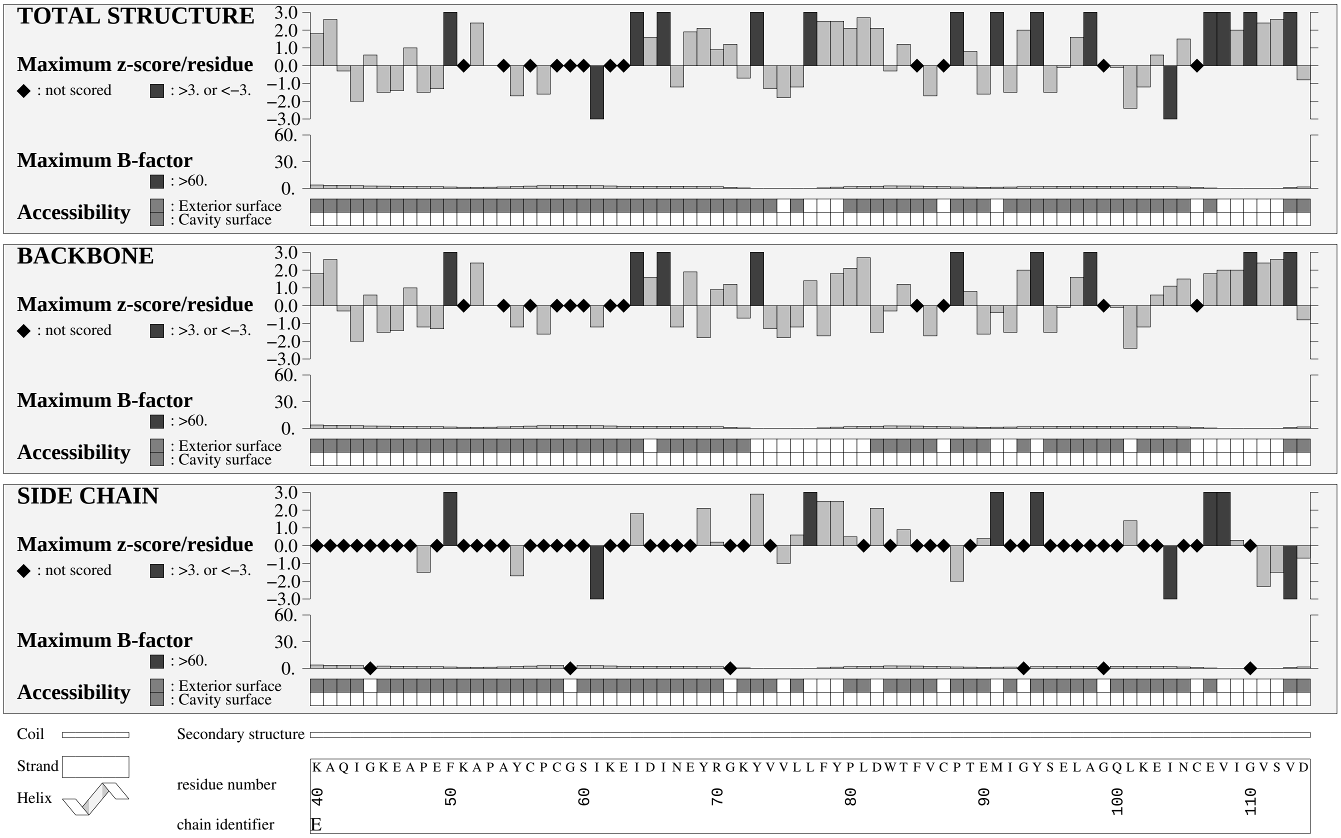

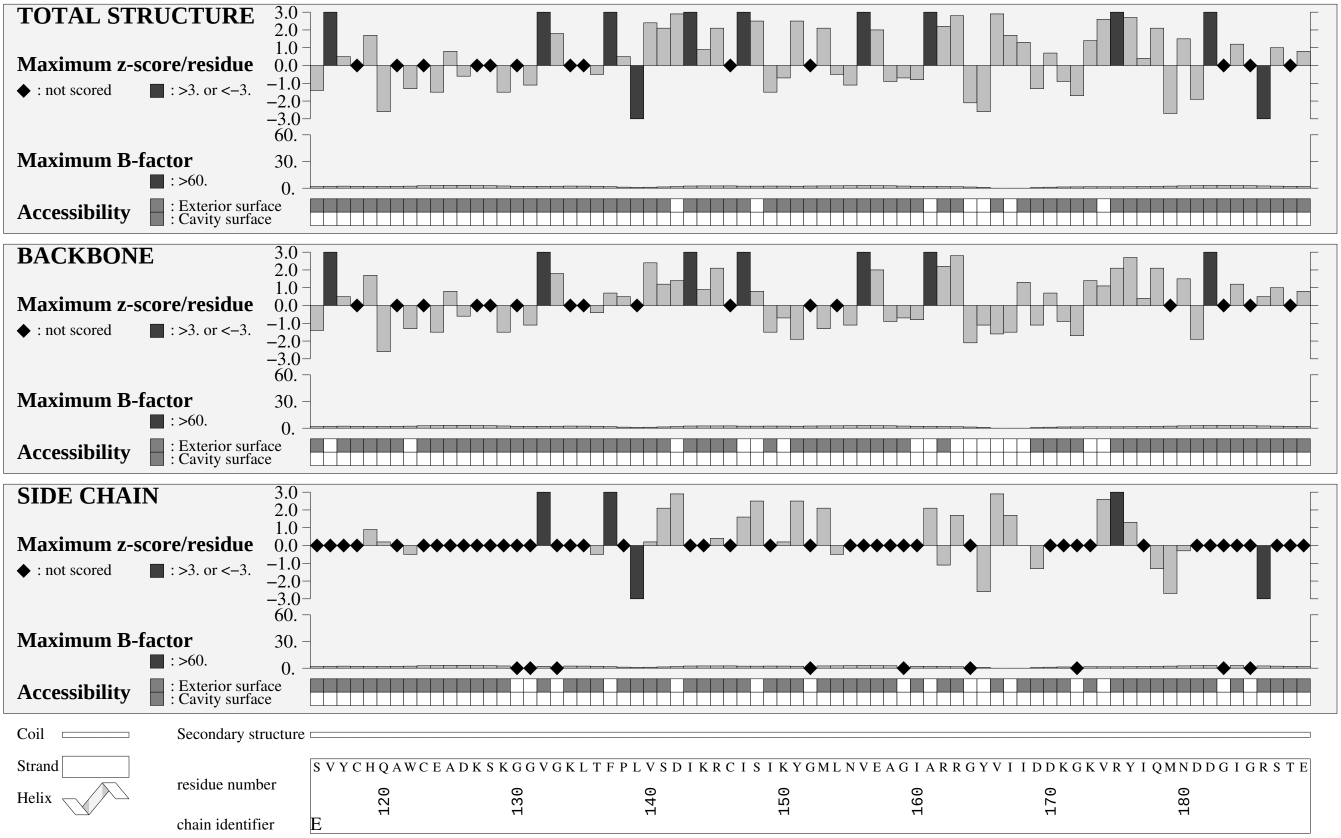

PROVE

Model_12.pdb

Analysis of residues (2)

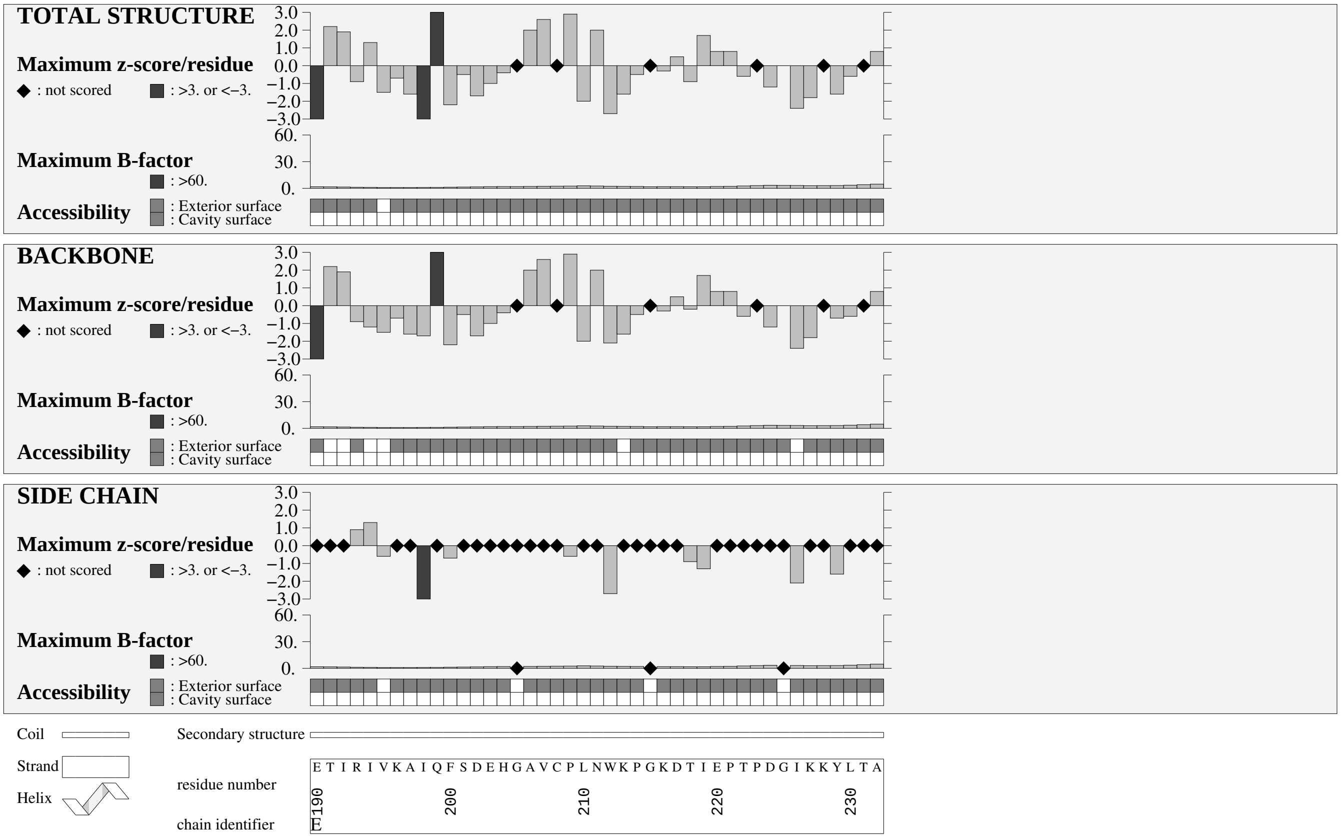

Supplement: Supplemental Information 3 — By comparing the highly refined PDB-deposited structures, PROVE calculates the volume of the atom in the predicted structure using a statistical Z-score. [file peerj-05-3160-s003.pdf]
