# Supplementary material for: In-silico prediction and modeling of the Entamoeba histolytica proteins: Serine-rich Entamoeba histolytica protein and 29 kDa Cysteine-rich protease
Source: PeerJ. 2017 Jun 28;5:e3160. doi: 10.7717/peerj.3160 (PMC5493030; doi:10.7717/peerj.3160)
Supplement: Supplemental Information 4 — Stereochemical properties of the proteins are checked by analysing the overall and residue-by-residue geometry [file peerj-05-3160-s004.pdf]

Chi1-Chi2 plots

23_128_72

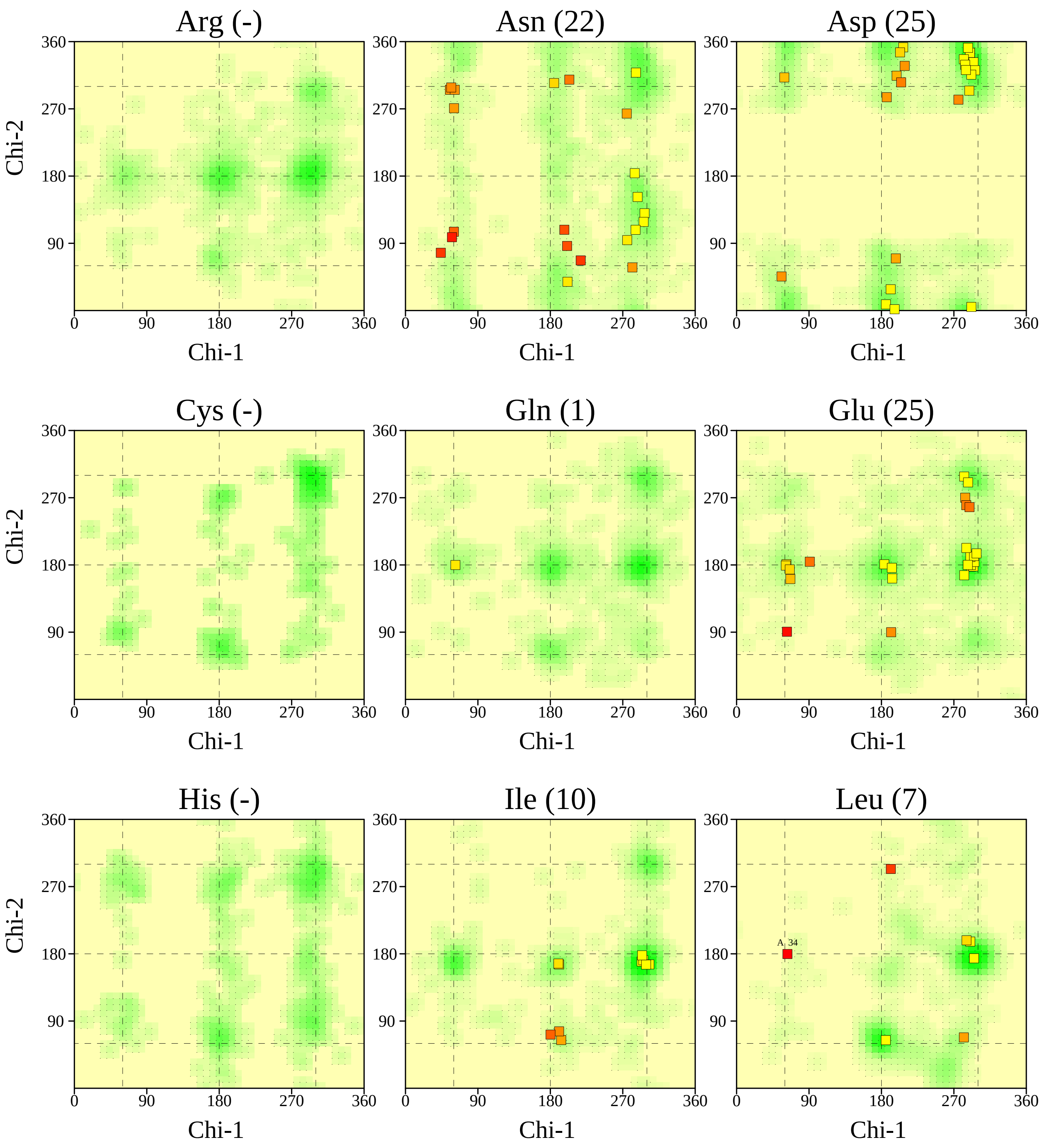

Numbers of residues are shown in brackets. Those in unfavourable conformations (score < -3.00) are labelled.
Shading shows favourable conformations as obtained from an analysis of 163 structures at resolution 2.0A or better.

Chi1-Chi2 plots

23_128_72

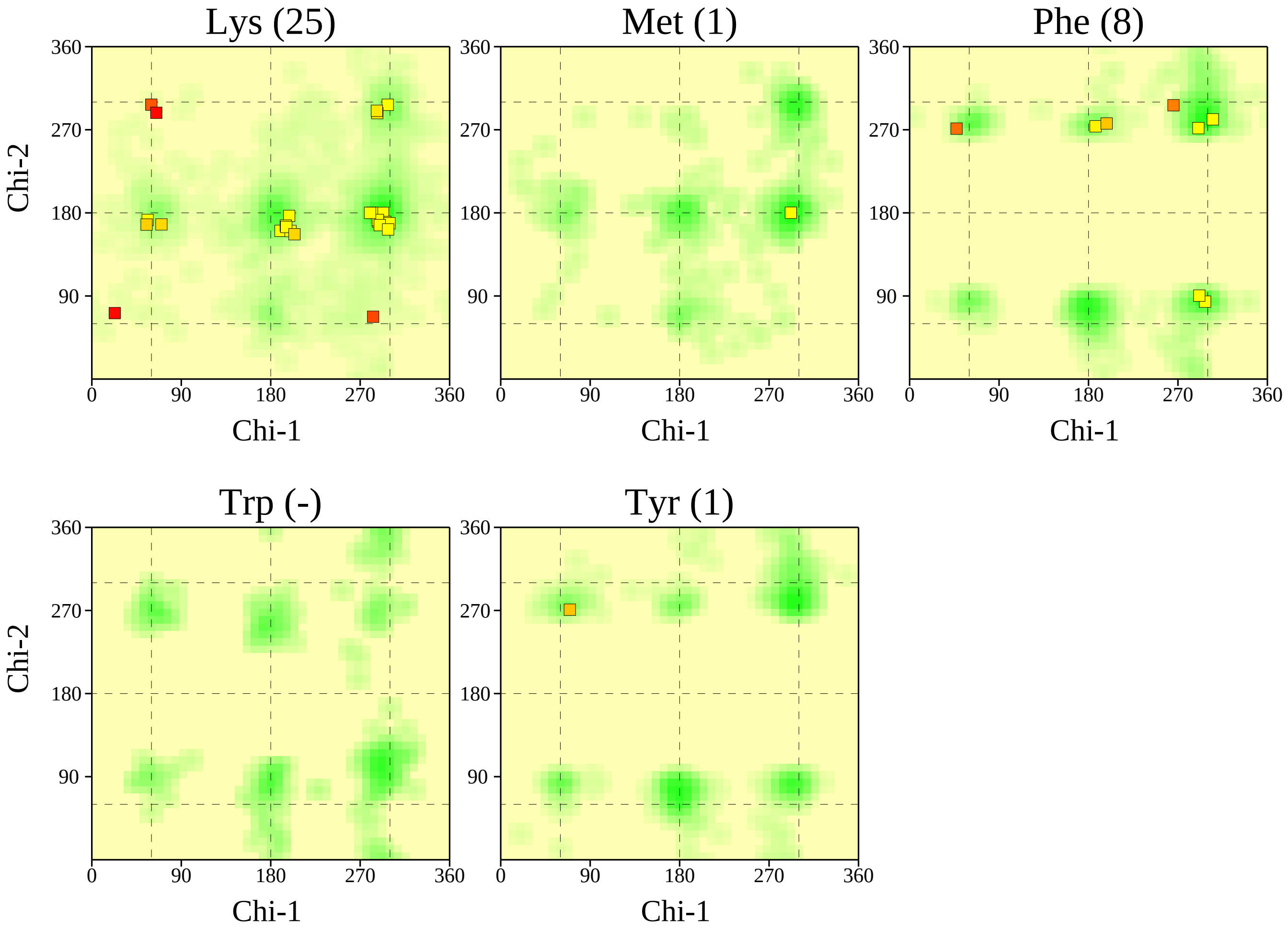

Numbers of residues are shown in brackets. Those in unfavourable conformations (score < -3.00) are labelled.
Shading shows favourable conformations as obtained from an analysis of 163 structures at resolution 2.0A or better.

Chi1-Chi2 plots

Model_12

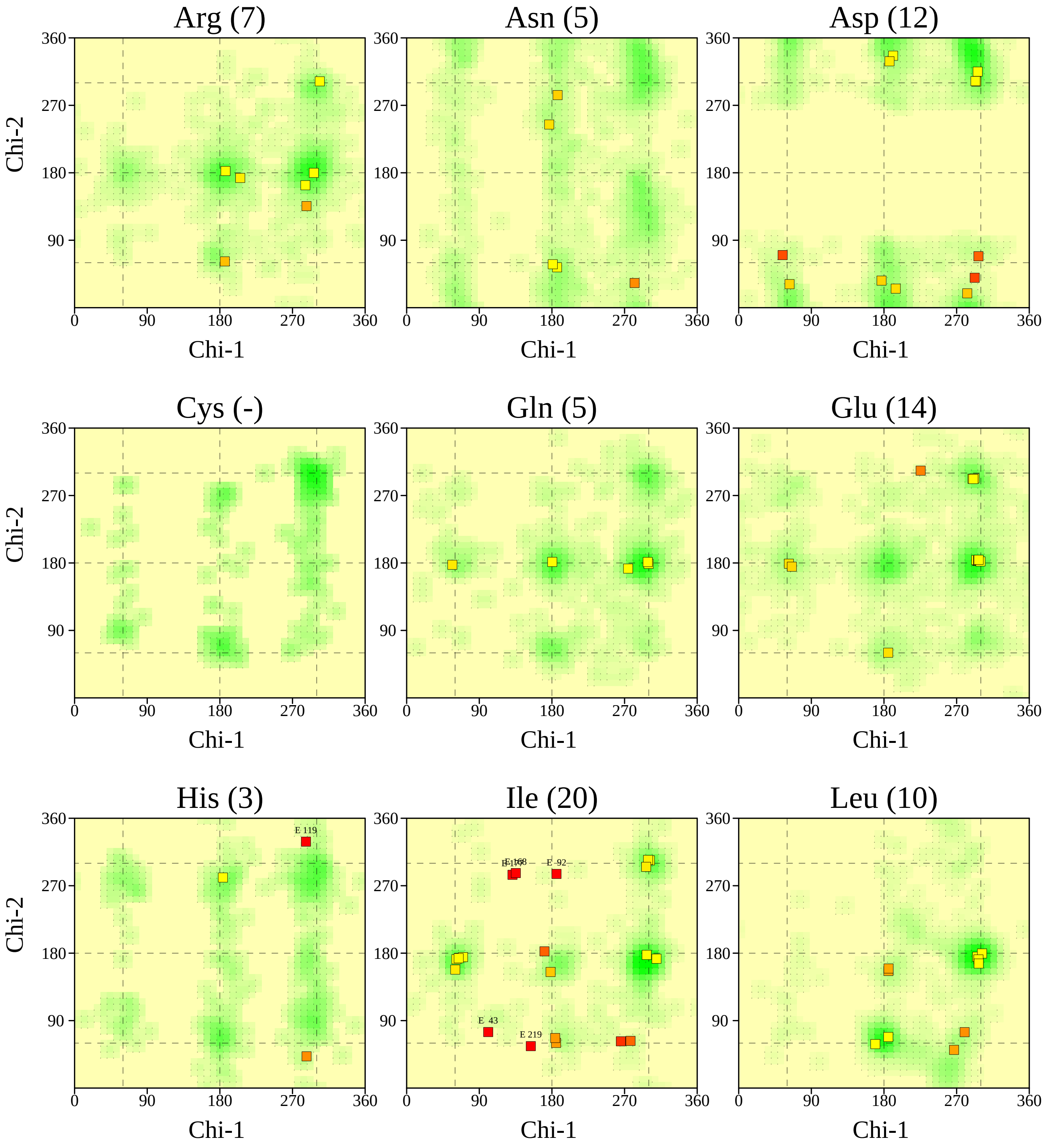

Numbers of residues are shown in brackets. Those in unfavourable conformations (score < -3.00) are labelled.
Shading shows favourable conformations as obtained from an analysis of 163 structures at resolution 2.0A or better.

Chi1-Chi2 plots

Model_12

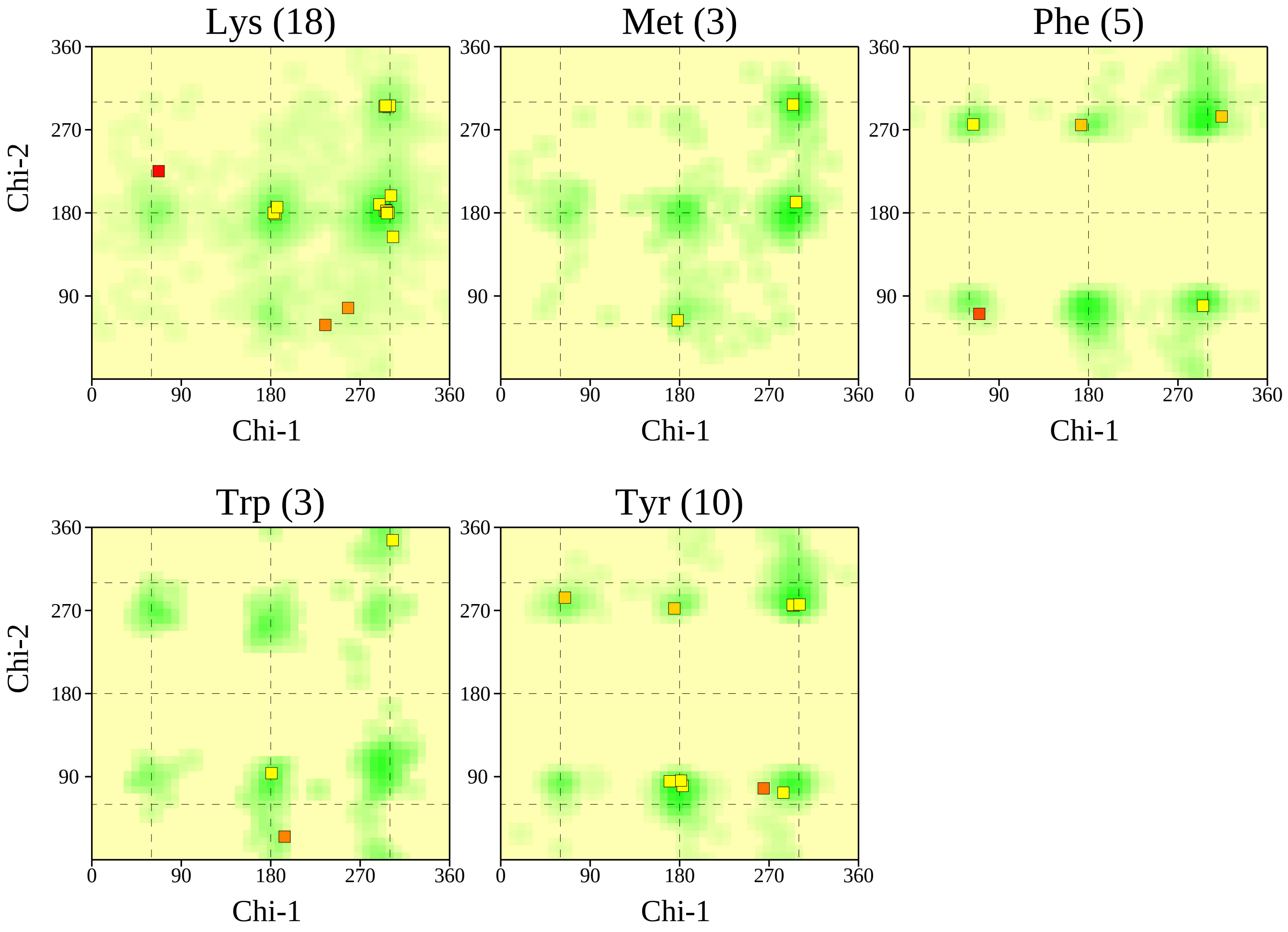

Numbers of residues are shown in brackets. Those in unfavourable conformations (score < -3.00) are labelled.
Shading shows favourable conformations as obtained from an analysis of 163 structures at resolution 2.0A or better.

Distorted geometry

23_128_72

Main-chain bond lengths

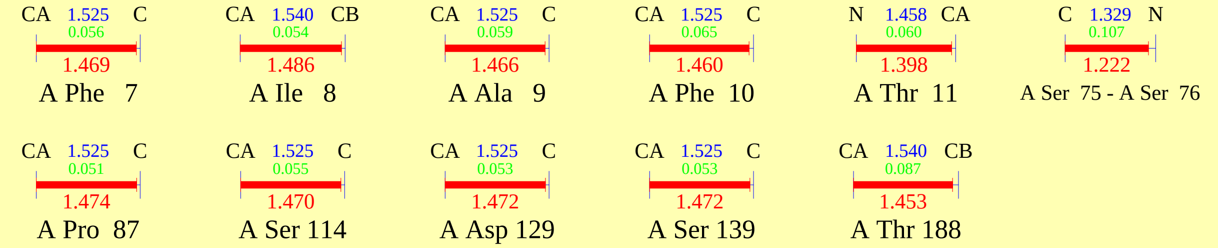

Bonds differing by > 0.05A from small-molecule values. Values shown: "ideal", difference, actual

Main-chain bond angles

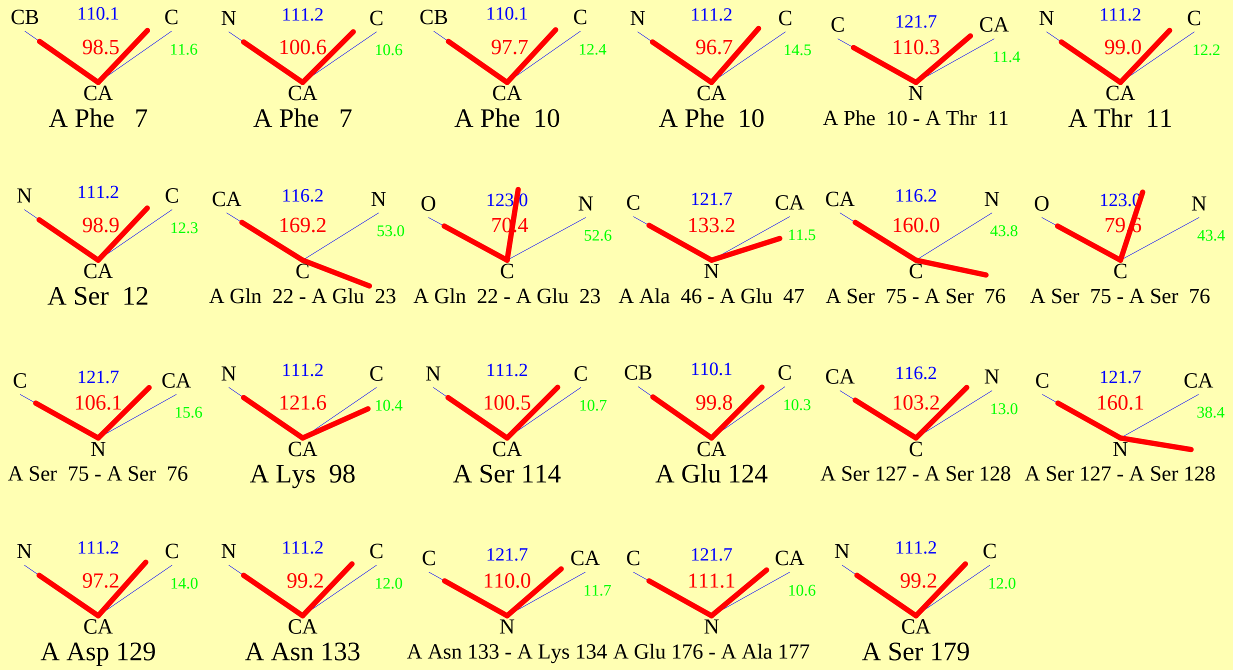

Bond angles differing by > 10.0 degrees from small-molec values. Values shown: "ideal", actual, diff.

Distorted geometry
Model_12

Main-chain bond lengths

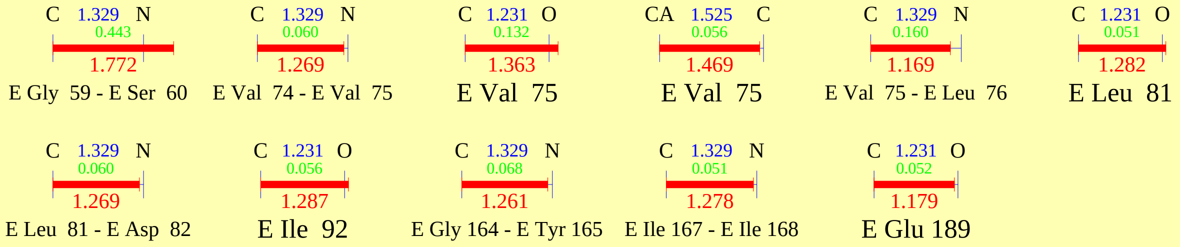

Bonds differing by > 0.05A from small-molecule values. Values shown: "ideal", difference, actual

Main-chain bond angles

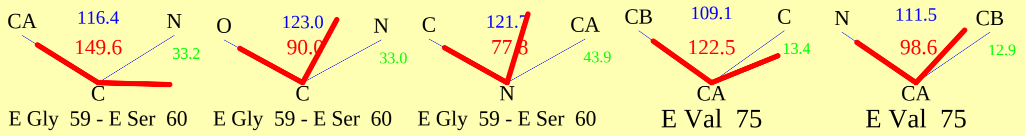

Bond angles differing by > 10.0 degrees from small-molec values. Values shown: "ideal", actual, diff.

Planar groups

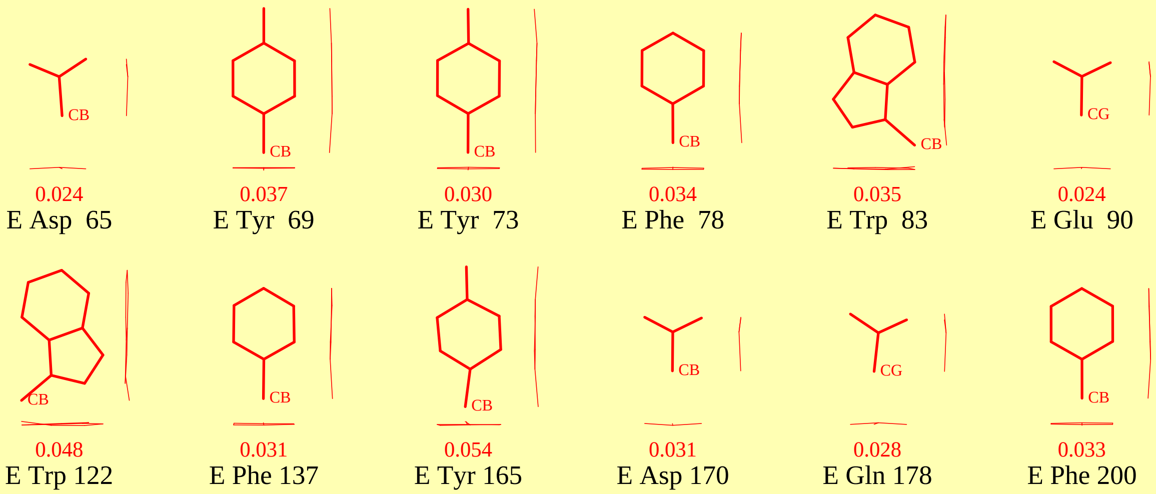

Distorted geometry
Model_12

Planar groups (contd)

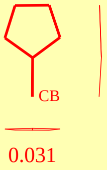

E His 204

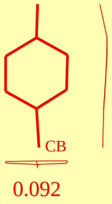

E Tyr 229

Sidechains with RMS dist. from planarity > 0.03A for rings, or > 0.02A otherwise. Value shown is RMS dist.
