# Supplementary material for: In-silico prediction and modeling of the Entamoeba histolytica proteins: Serine-rich Entamoeba histolytica protein and 29 kDa Cysteine-rich protease
Source: PeerJ. 2017 Jun 28;5:e3160. doi: 10.7717/peerj.3160 (PMC5493030; doi:10.7717/peerj.3160)
Supplement: Supplemental Information 5 — The graphs on the main-chain parameters plot show how the structure compares with well-refined structures at a similar resolution. The dark band represents the results from the well-refined structures; the central line is a least-squares fit to the mean trend as a function of resolution, while the width of the band on either side of it corresponds to a variation of one standard deviation about the mean. [file peerj-05-3160-s005.pdf]

Main-chain bond angles

23_128_72

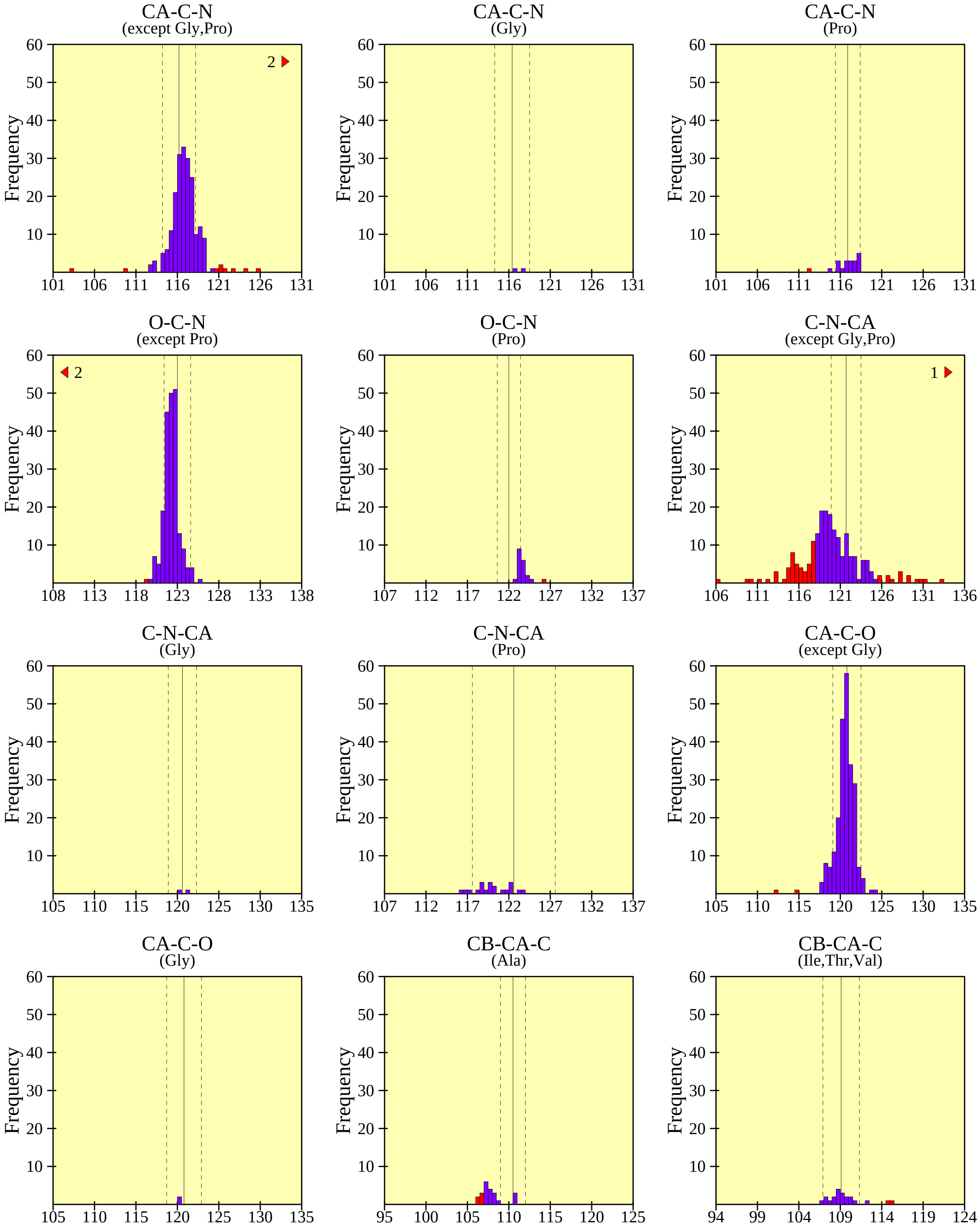

Black bars > 2.0 st. devs. from mean. ◀ or ▶ signifies data points off the graph in the direction shown.
Solid and dashed lines represent the mean and standard deviation values as per Engh & Huber small-molecule data.

Main-chain bond angles

23_128_72

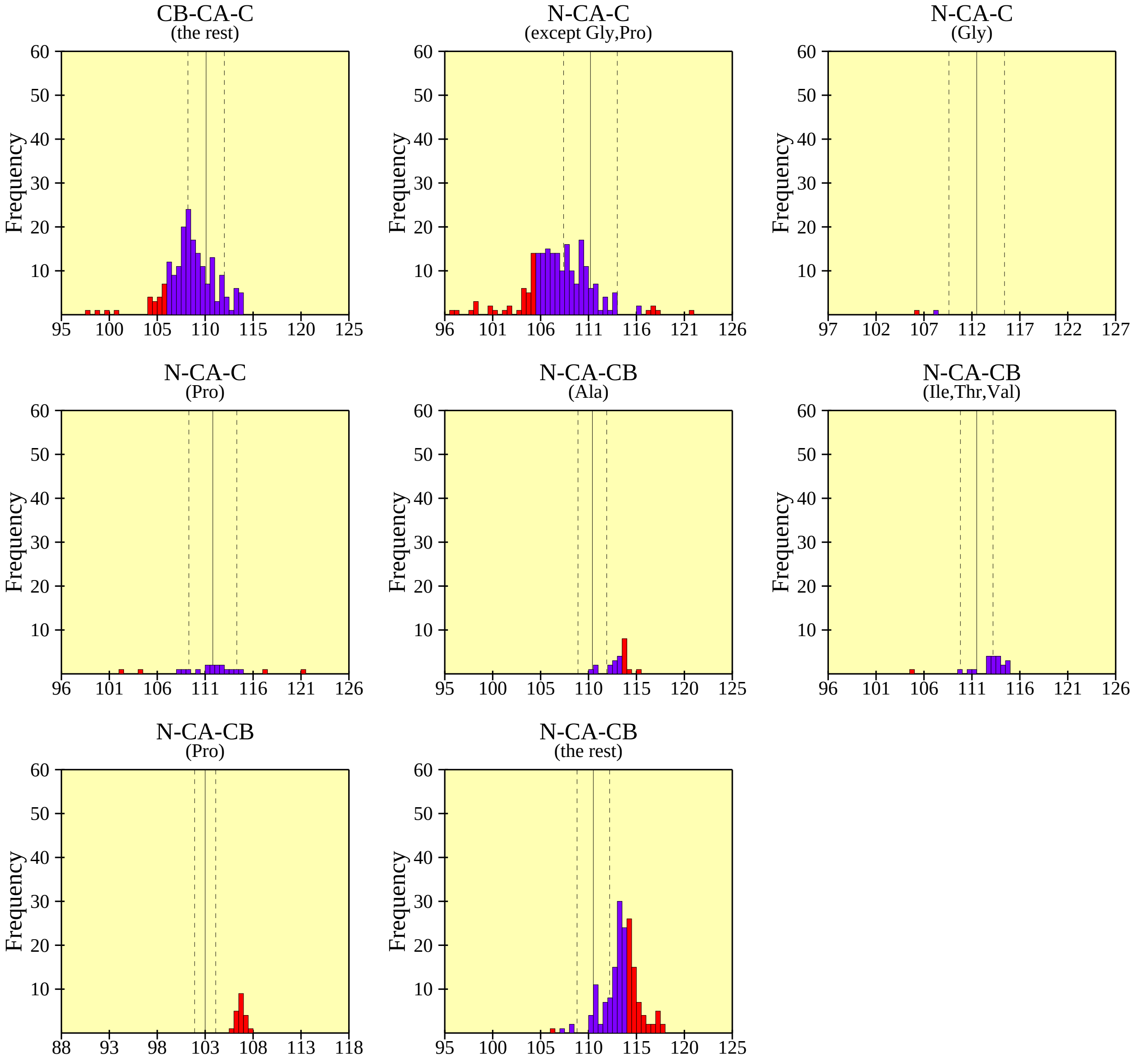

Black bars > 2.0 st. devs. from mean.

Solid and dashed lines represent the mean and standard deviation values as per Engh & Huber small-molecule data.

Main-chain bond angles

Model_12

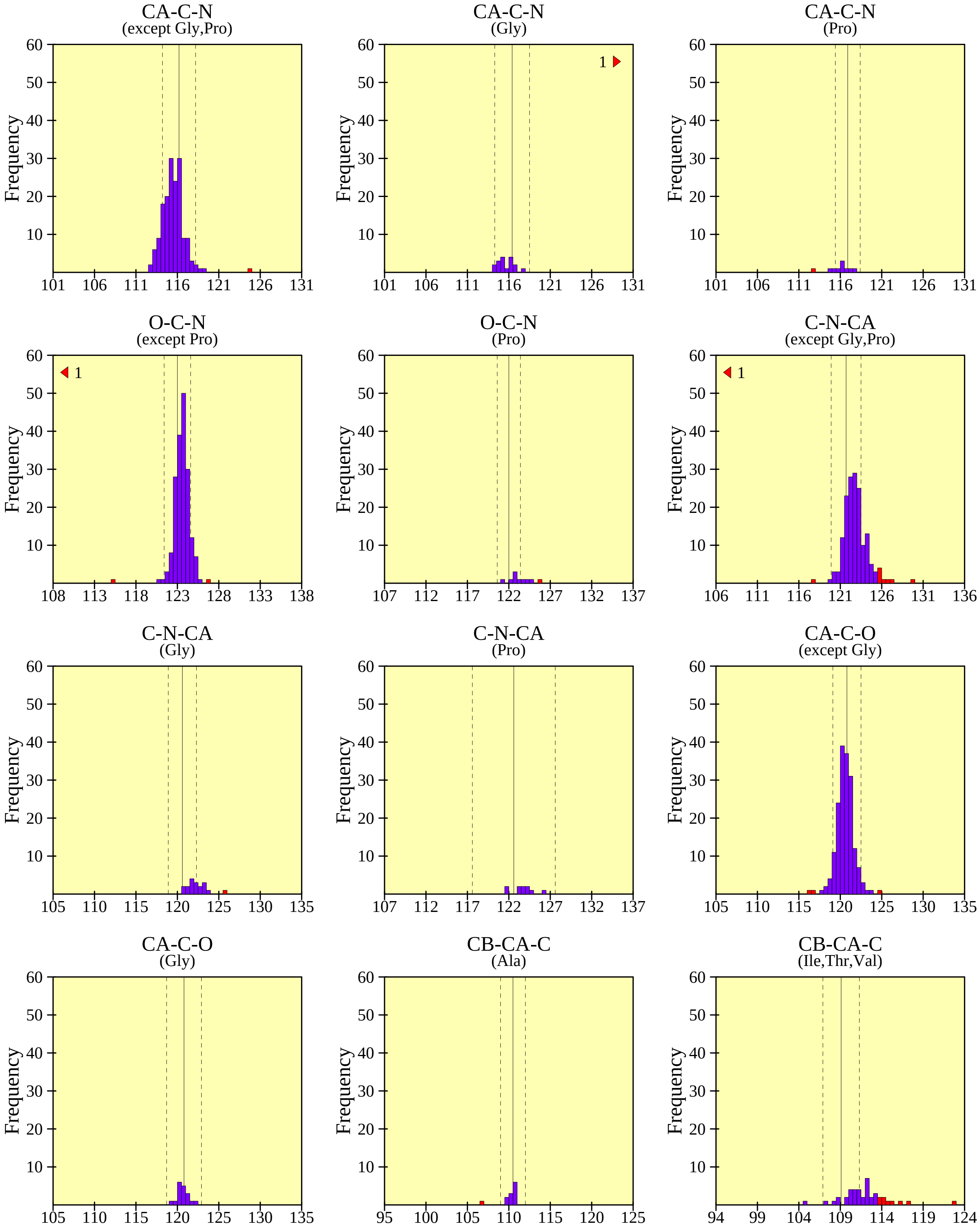

Black bars > 2.0 st. devs. from mean. ◀ or ▶ signifies data points off the graph in the direction shown.
Solid and dashed lines represent the mean and standard deviation values as per Engh & Huber small-molecule data.

Main-chain bond angles

Model_12

CB-CA-C
(the rest)

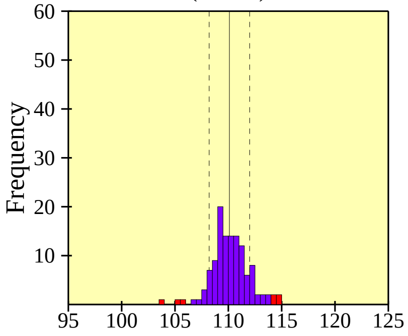

N-CA-C
(except Gly,Pro)

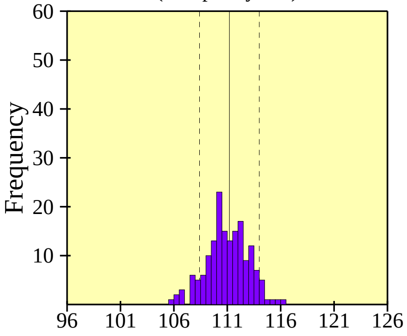

N-CA-C
(Gly)

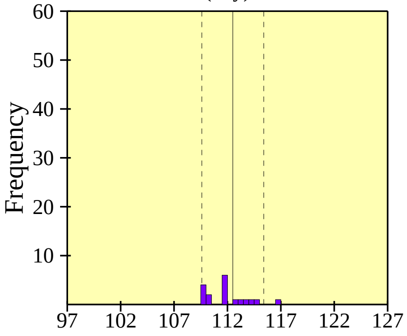

N-CA-C
(Pro)

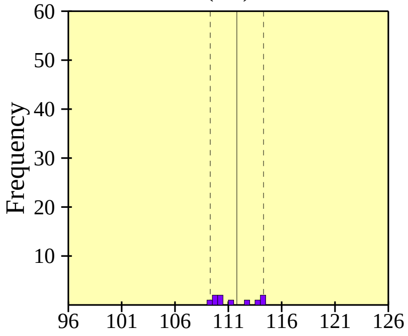

N-CA-CB
(Ala)

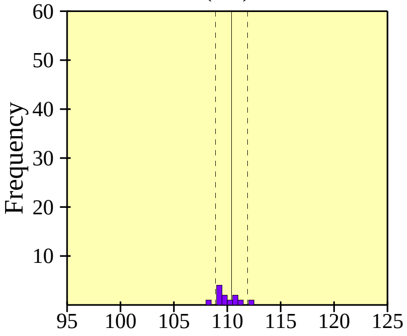

N-CA-CB
(Ile,Thr,Val)

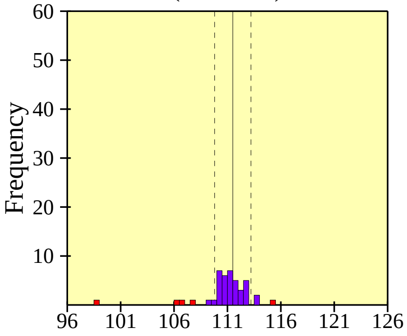

N-CA-CB
(Pro)

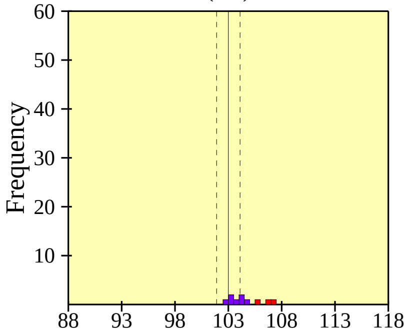

N-CA-CB
(the rest)

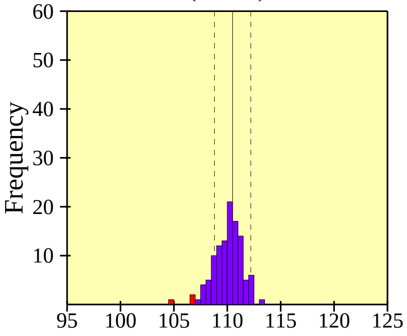

Black bars > 2.0 st. devs. from mean.

Solid and dashed lines represent the mean and standard deviation values as per Engh & Huber small-molecule data.

Main-chain bond lengths

23_128_72

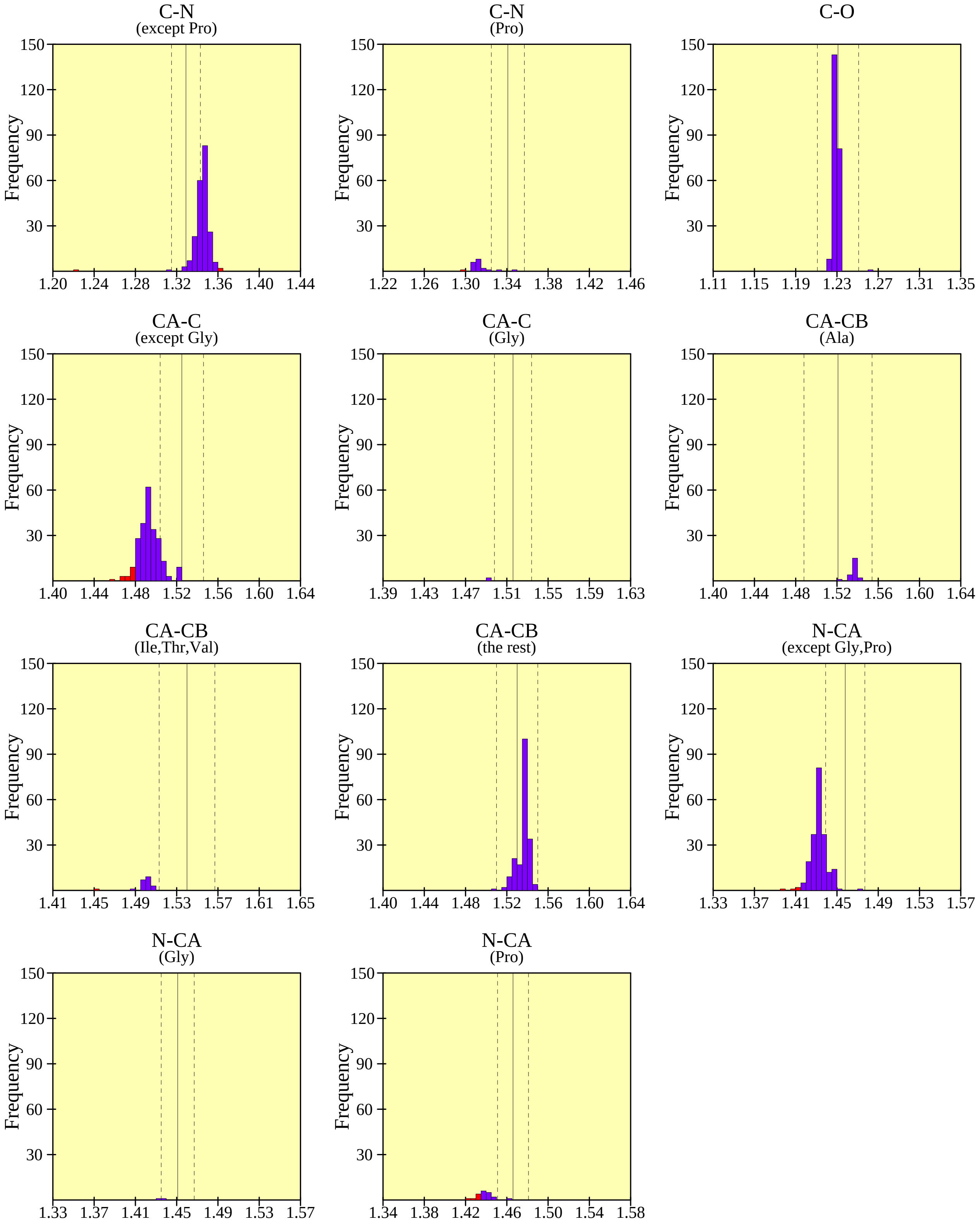

Black bars > 2.0 st. devs. from mean.

Solid and dashed lines represent the mean and standard deviation values as per Engh & Huber small-molecule data.

Main-chain bond lengths

Model_12

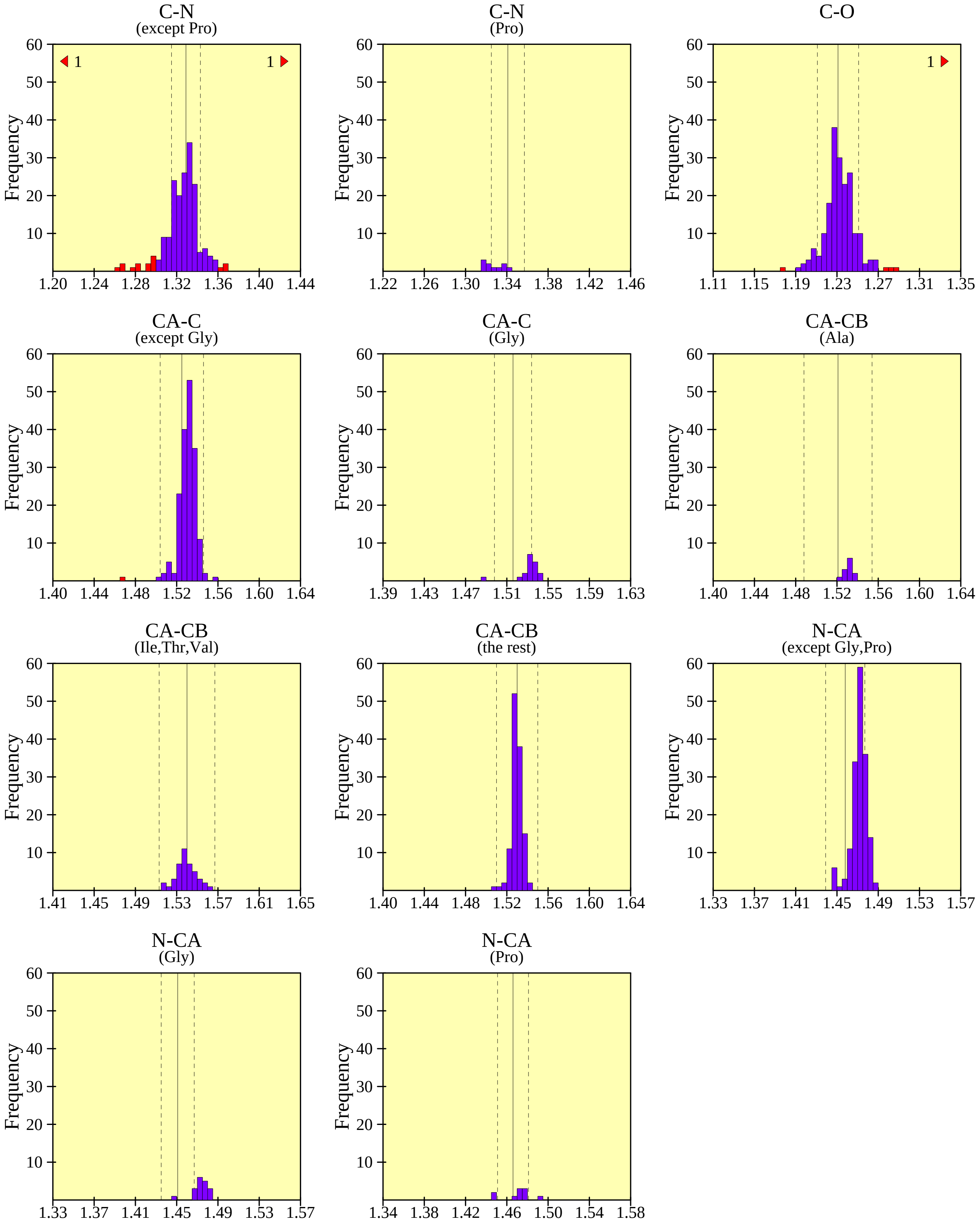

Black bars > 2.0 st. devs. from mean. ◀ or ▶ signifies data points off the graph in the direction shown.
Solid and dashed lines represent the mean and standard deviation values as per Engh & Huber small-molecule data.

PROCHECK

Main-chain parameters

23_128_72

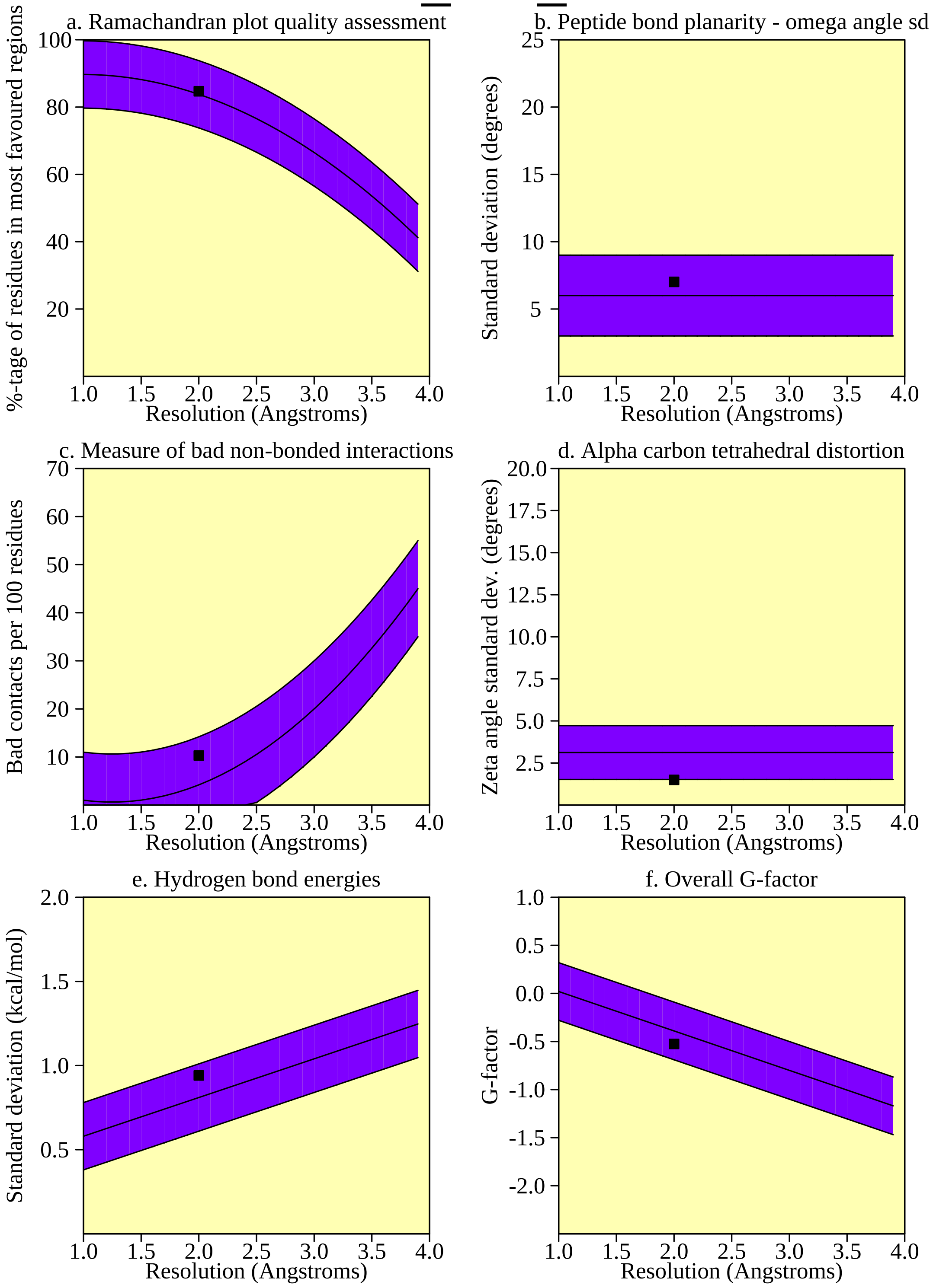

Plot statistics

Stereochemical parameter	No. of data pts	Parameter value	Comparison values Typical value	Band width	No. of band widths from mean	
a. %-tage residues in A, B, L	209	84.7	83.8	10.0	0.1	Inside
b. Omega angle st dev	232	7.0	6.0	3.0	0.3	Inside
c. Bad contacts / 100 residues	24	10.3	4.2	10.0	0.6	Inside
d. Zeta angle st dev	231	1.5	3.1	1.6	-1.0	BETTER
e. H-bond energy st dev	89	0.9	0.8	0.2	0.7	Inside
f. Overall G-factor	233	-0.5	-0.4	0.3	-0.5	Inside

PROCHECK

Main-chain parameters

Model_12

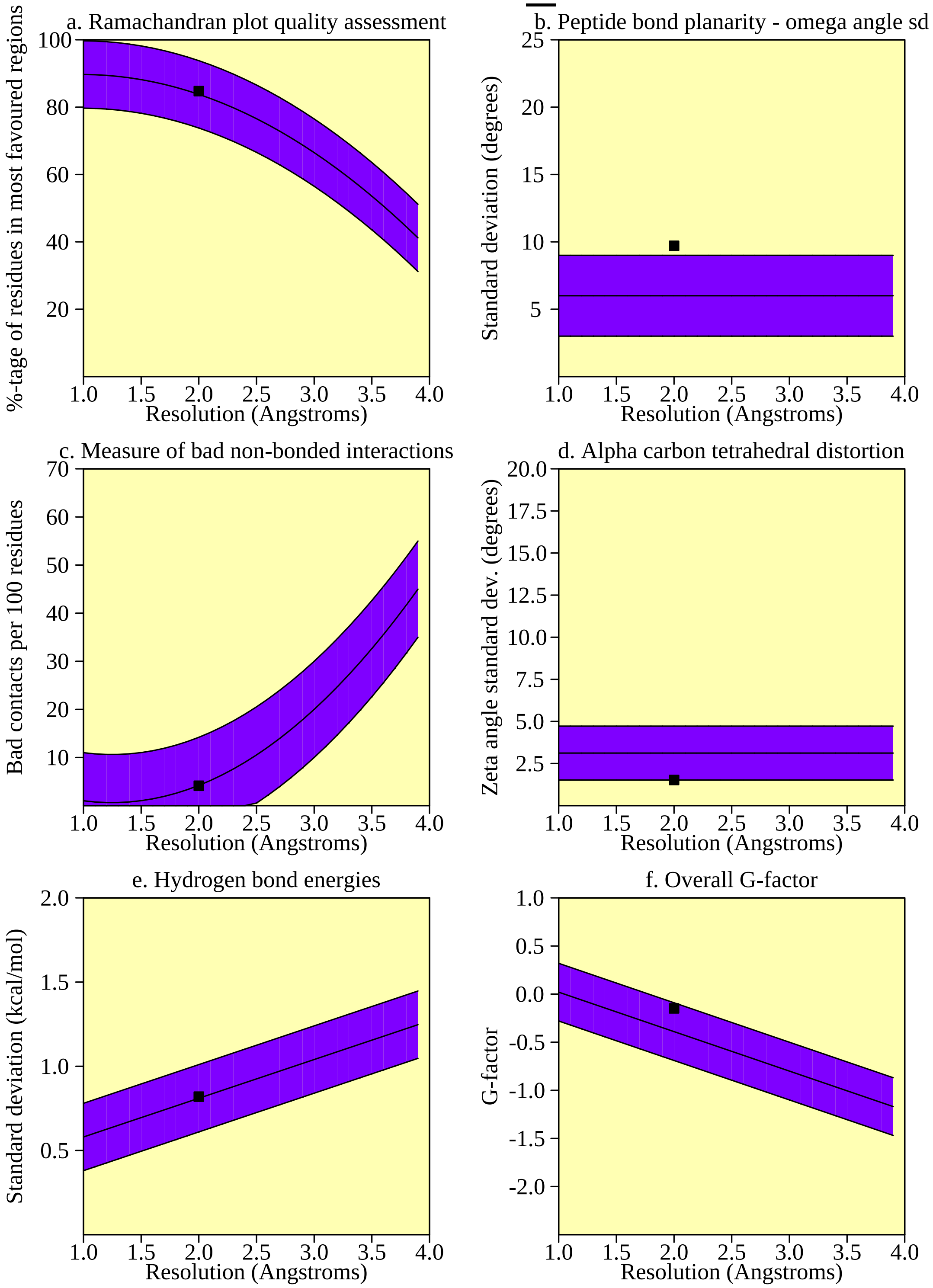

Plot statistics

Stereochemical parameter	No. of data pts	Parameter value	Comparison values: Typical value	Comparison values: Band width	No. of band widths from mean	
a. %-tage residues in A, B, L	164	84.8	83.8	10.0	0.1	Inside
b. Omega angle st dev	193	9.7	6.0	3.0	1.2	WORSE
c. Bad contacts / 100 residues	8	4.1	4.2	10.0	0.0	Inside
d. Zeta angle st dev	176	1.5	3.1	1.6	-1.0	Inside
e. H-bond energy st dev	118	0.8	0.8	0.2	0.0	Inside
f. Overall G-factor	194	-0.1	-0.4	0.3	0.8	Inside
