# Supplementary material for: In-silico prediction and modeling of the Entamoeba histolytica proteins: Serine-rich Entamoeba histolytica protein and 29 kDa Cysteine-rich protease
Source: PeerJ. 2017 Jun 28;5:e3160. doi: 10.7717/peerj.3160 (PMC5493030; doi:10.7717/peerj.3160)
Supplement: Supplemental Information 6 — The 5 graphs in the side-chain parameters consider the following properties for each protein.1. Standard deviation of the chi-1 gauche minus torsion angles. 2. Standard deviation of the chi-1 trans torsion angles. 3. Standard deviation of the chi-1 gauche plus torsion angles. 4. Pooled standard deviation of all chi-1 torsion angles. 5. Standard deviation of the chi-2 trans torsion angles. [file peerj-05-3160-s006.pdf]

PROCHECK

Side-chain parameters

23_128_72

a. Chi-1 gauche minus

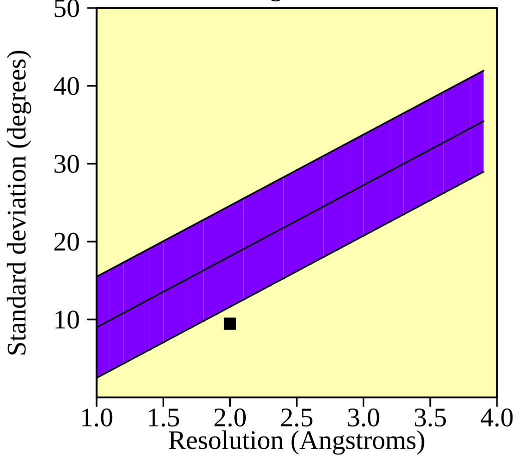

b. Chi-1 trans

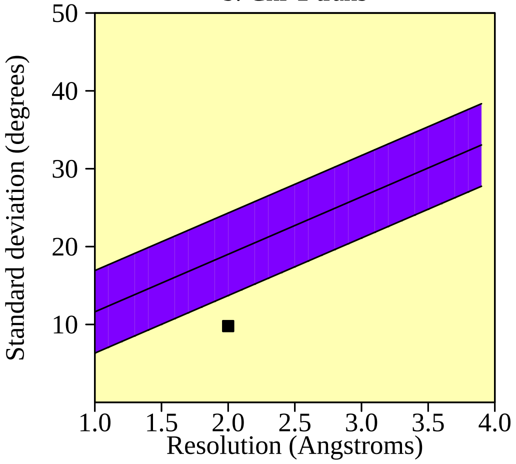

c. Chi-1 gauche plus

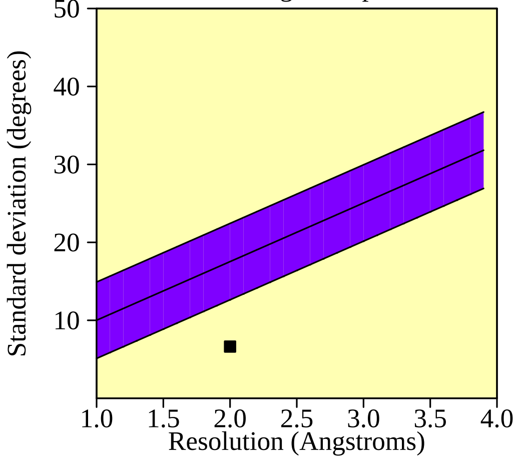

d. Chi-1 pooled standard deviation

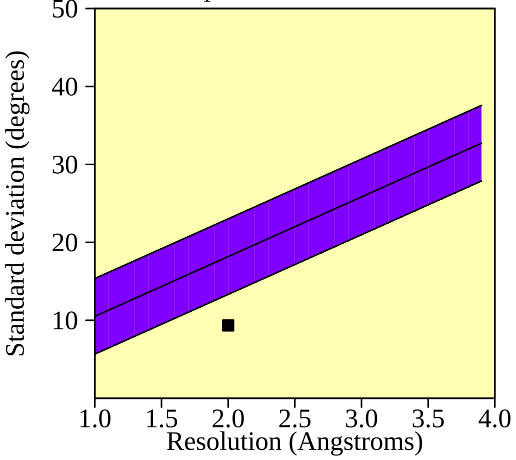

e. Standard deviation of Chi-2 trans angle

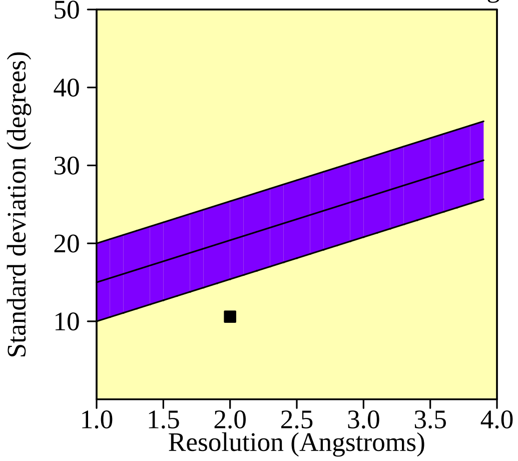

23_128_72

Plot statistics

Stereochemical parameter	No. of data pts	Parameter value	Comparison values: Typical value	Comparison values: Band width	No. of band widths from mean	
a. Chi-1 gauche minus st dev	42	9.5	18.1	6.5	-1.3	BETTER
b. Chi-1 trans st dev	54	9.8	19.0	5.3	-1.7	BETTER
c. Chi-1 gauche plus st dev	93	6.6	17.5	4.9	-2.2	BETTER
d. Chi-1 pooled st dev	189	9.3	18.2	4.8	-1.8	BETTER
e. Chi-2 trans st dev	49	10.6	20.4	5.0	-2.0	BETTER

PROCHECK

Side-chain parameters

Model_12

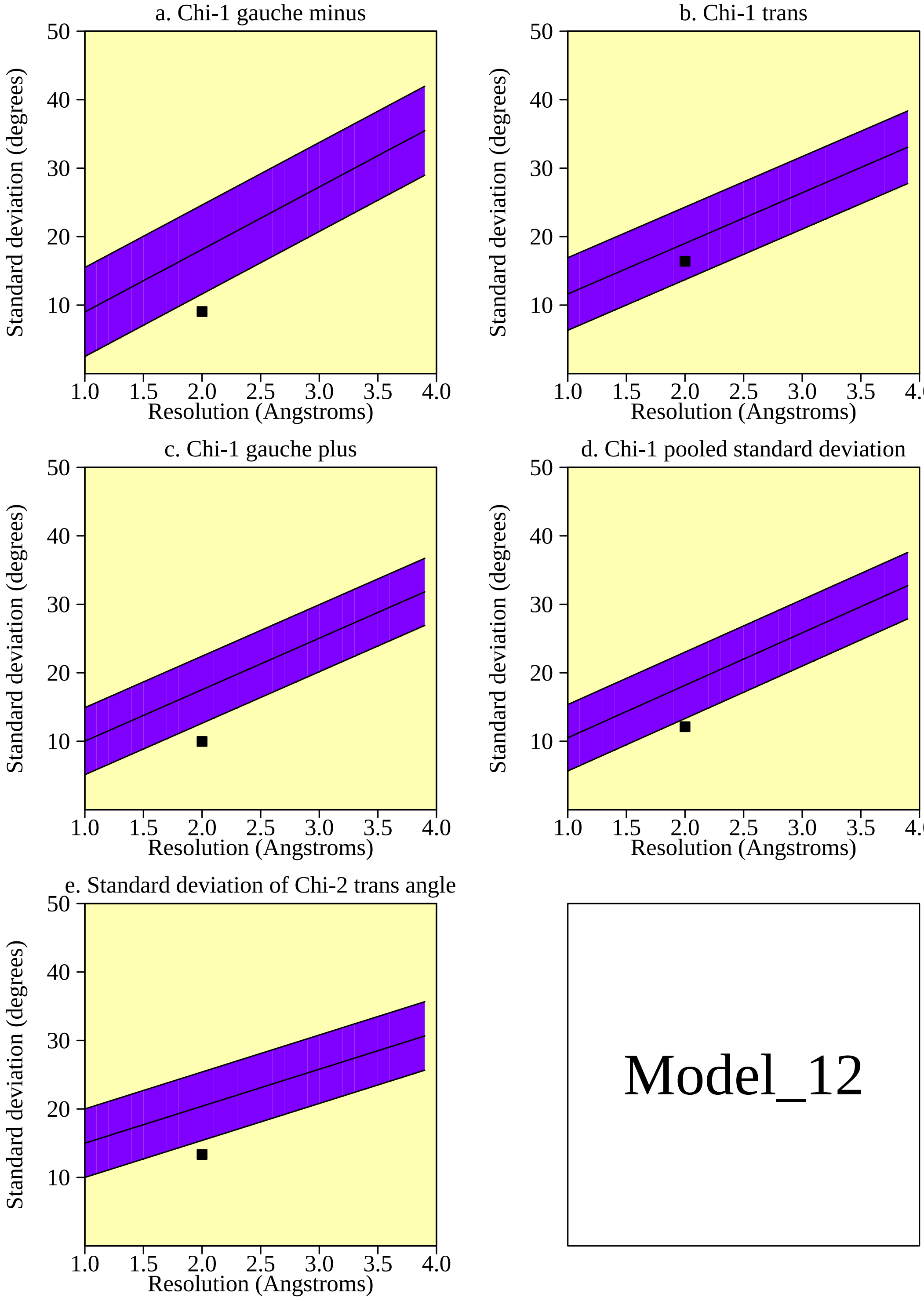

Plot statistics

Stereochemical parameter	No. of data pts	Parameter value	Comparison values Typical value	Band width	No. of band widths from mean	
a. Chi-1 gauche minus st dev	30	9.1	18.1	6.5	-1.4	BETTER
b. Chi-1 trans st dev	54	16.4	19.0	5.3	-0.5	Inside
c. Chi-1 gauche plus st dev	70	10.0	17.5	4.9	-1.5	BETTER
d. Chi-1 pooled st dev	154	12.1	18.2	4.8	-1.2	BETTER
e. Chi-2 trans st dev	46	13.4	20.4	5.0	-1.4	BETTER
