# Supplementary material for: In-silico prediction and modeling of the Entamoeba histolytica proteins: Serine-rich Entamoeba histolytica protein and 29 kDa Cysteine-rich protease
Source: PeerJ. 2017 Jun 28;5:e3160. doi: 10.7717/peerj.3160 (PMC5493030; doi:10.7717/peerj.3160)
Supplement: Supplemental Information 7 — The PROCHECK performs post-structure analysis by completely analysing the individual residues and their interactions in the predicted structure. [file peerj-05-3160-s007.pdf]

PROCHECK

Residue properties

23_128_72

a. Absolute deviation from mean Chi-1 value (excl. Pro)

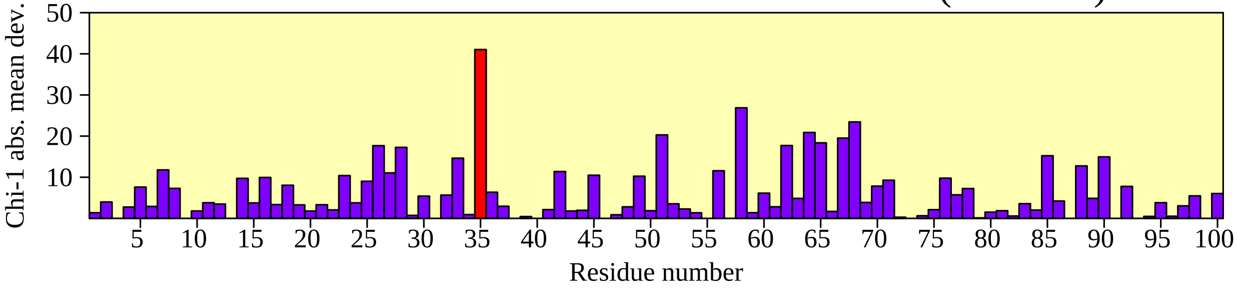

b. Absolute deviation from mean of omega torsion

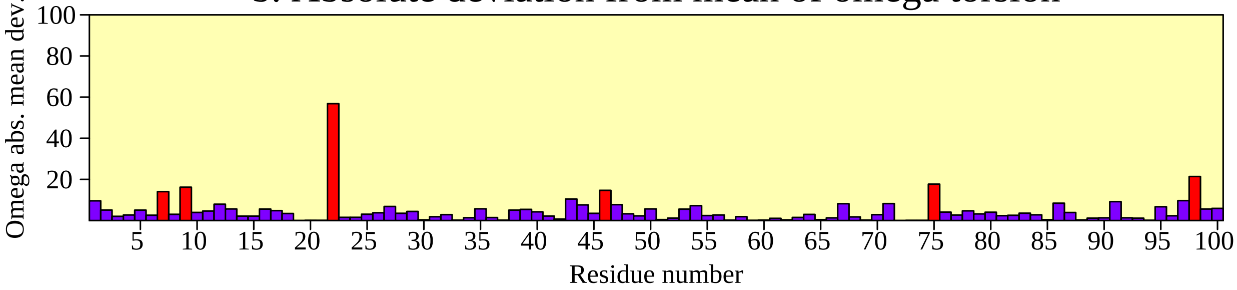

c. C-alpha chirality: abs. deviation of zeta torsion

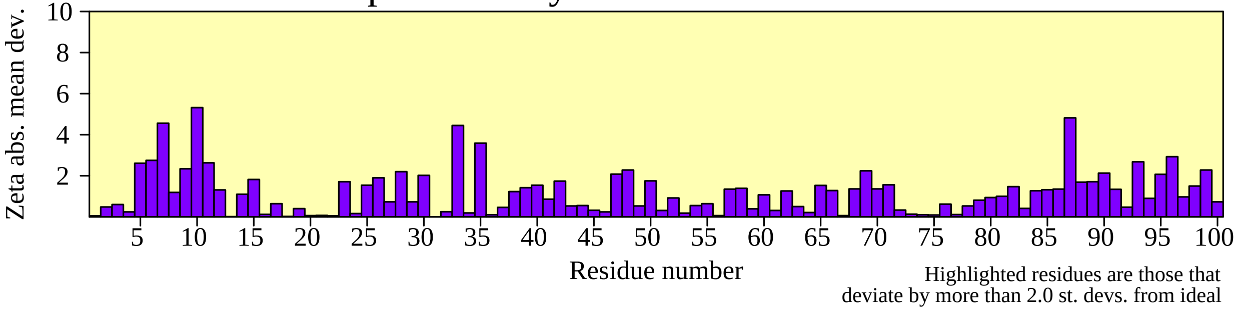

Highlighted residues are those that deviate by more than 2.0 st. devs. from ideal

d. Secondary structure & estimated accessibility

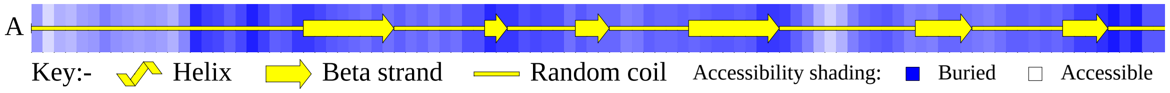

e. Sequence & Ramachandran regions

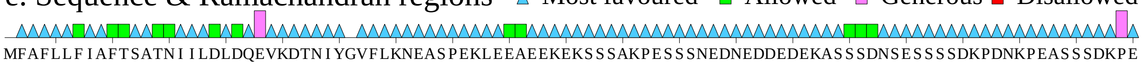

f. Max. deviation (see listing)

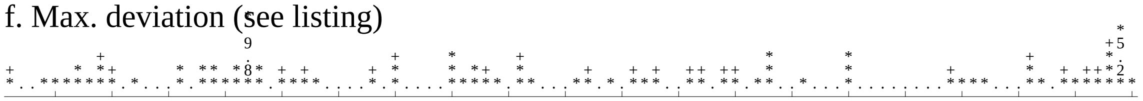

g. G-factors

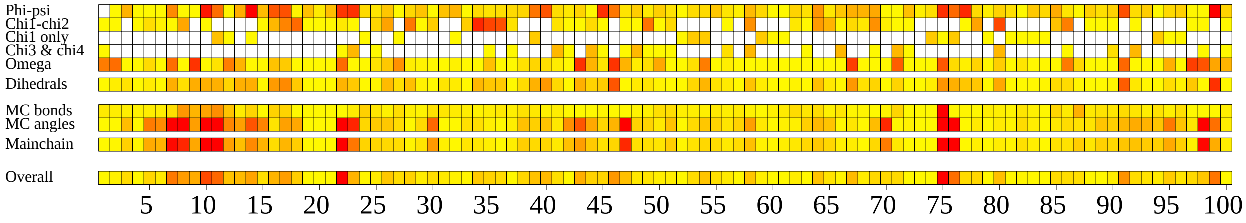

Residue properties

23_128_72

a. Absolute deviation from mean Chi-1 value (excl. Pro)

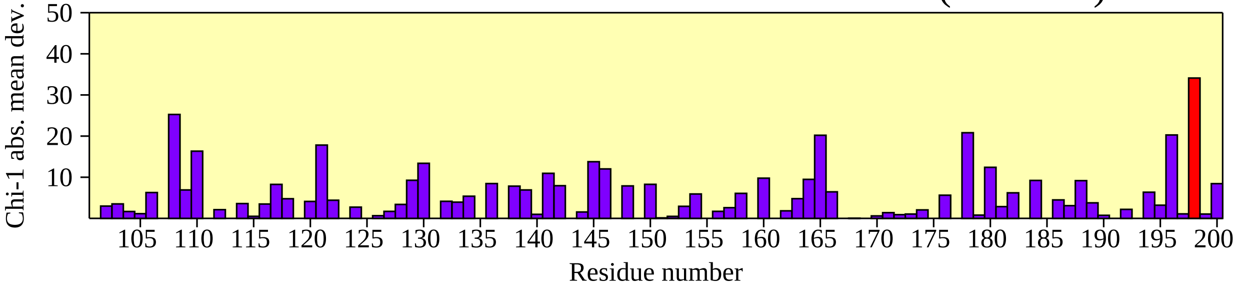

b. Absolute deviation from mean of omega torsion

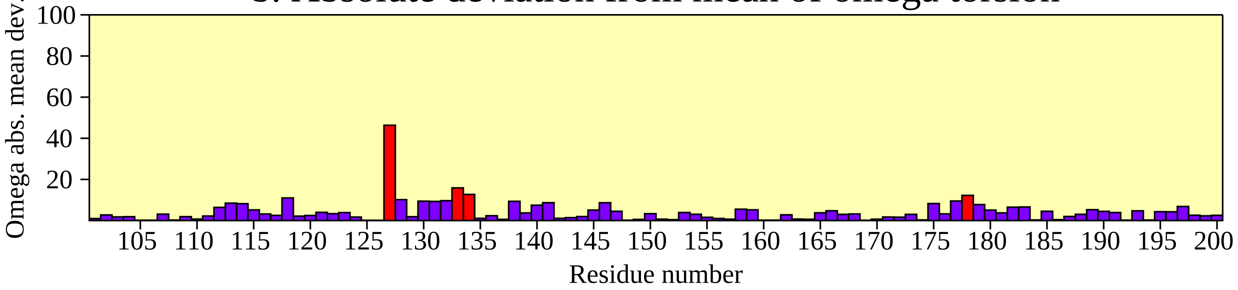

c. C-alpha chirality: abs. deviation of zeta torsion

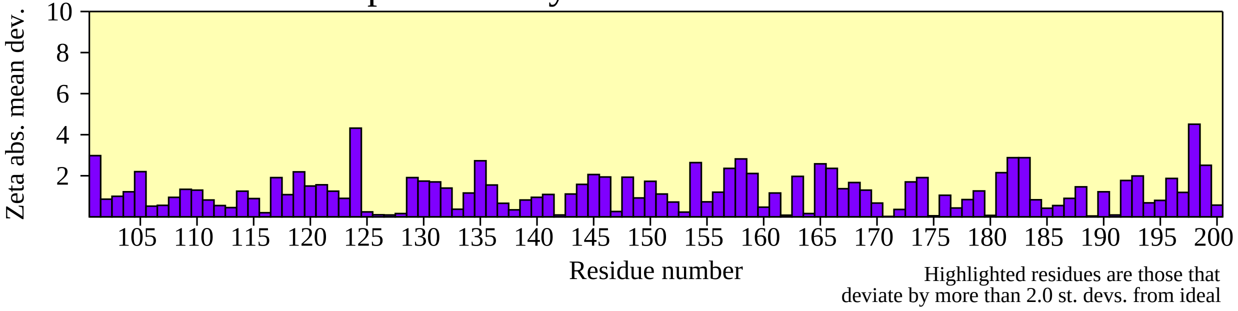

Highlighted residues are those that deviate by more than 2.0 st. devs. from ideal

d. Secondary structure & estimated accessibility

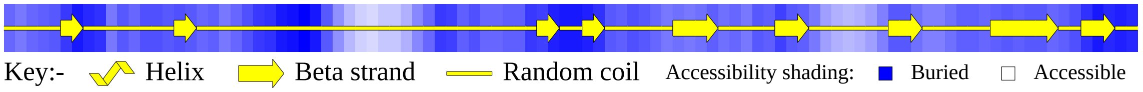

e. Sequence & Ramachandran regions

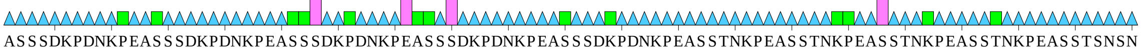

f. Max. deviation (see listing)

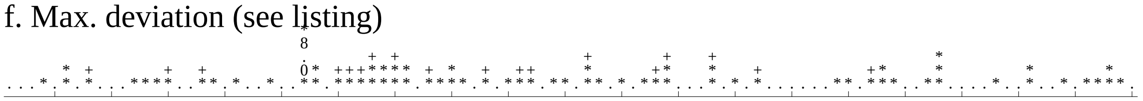

g. G-factors

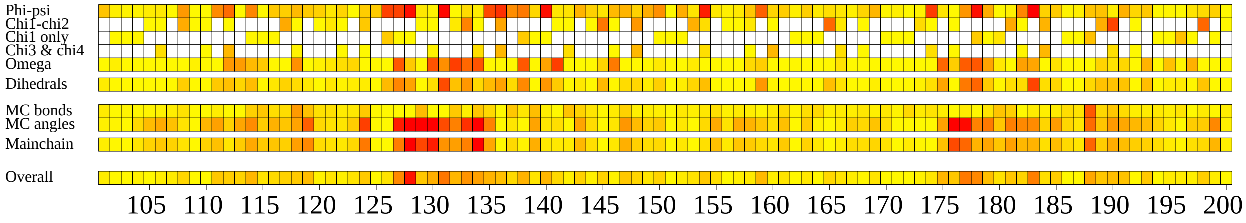

Residue properties

23_128_72

a. Absolute deviation from mean Chi-1 value (excl. Pro)

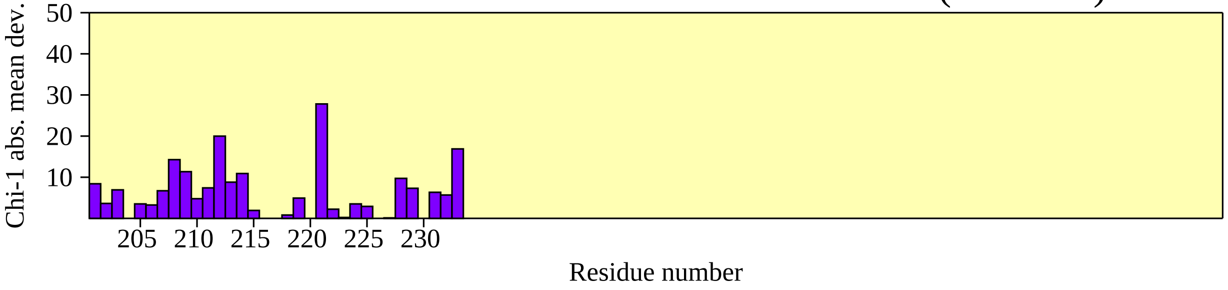

b. Absolute deviation from mean of omega torsion

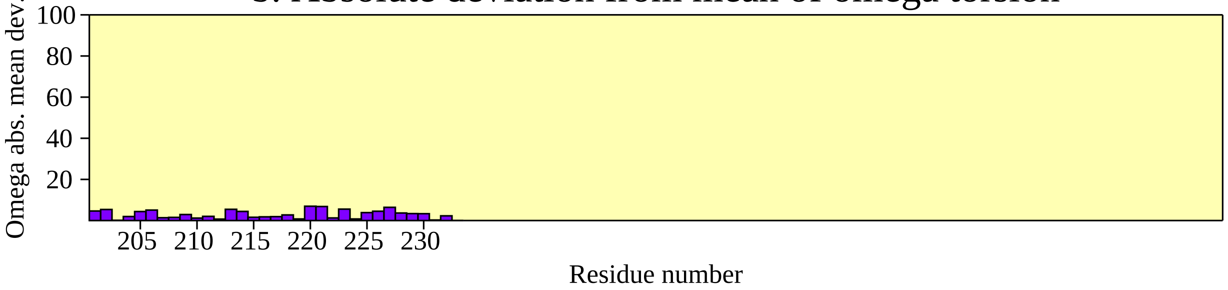

c. C-alpha chirality: abs. deviation of zeta torsion

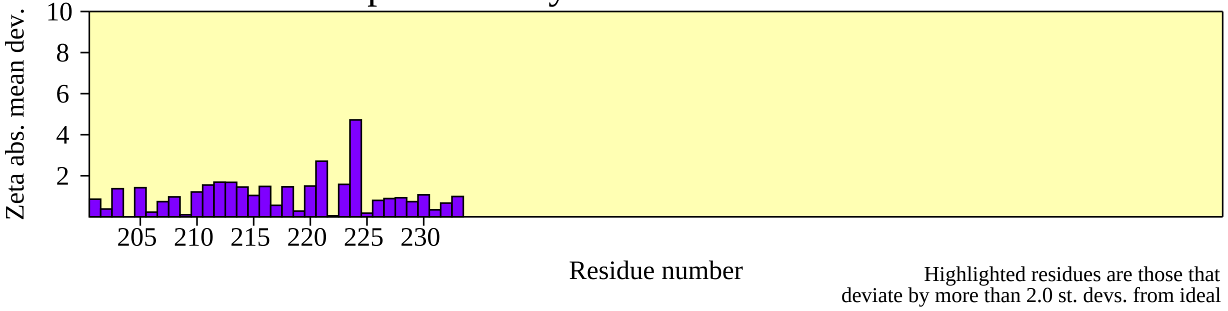

Highlighted residues are those that deviate by more than 2.0 st. devs. from ideal

d. Secondary structure & estimated accessibility

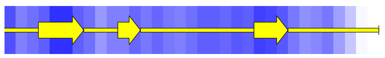

Key:- Helix Beta strand Random coil Accessibility shading: Buried Accessible

e. Sequence & Ramachandran regions

Most favoured Allowed Generous Disallowed

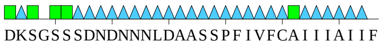

f. Max. deviation (see listing)

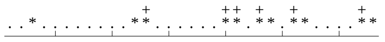

g. G-factors

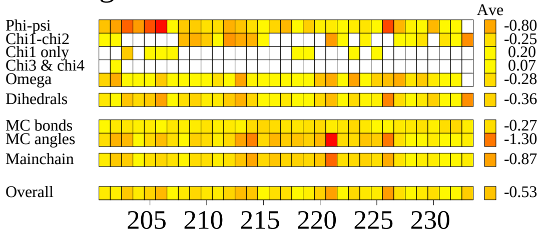

Residue properties
Model_12

a. Absolute deviation from mean Chi-1 value (excl. Pro)

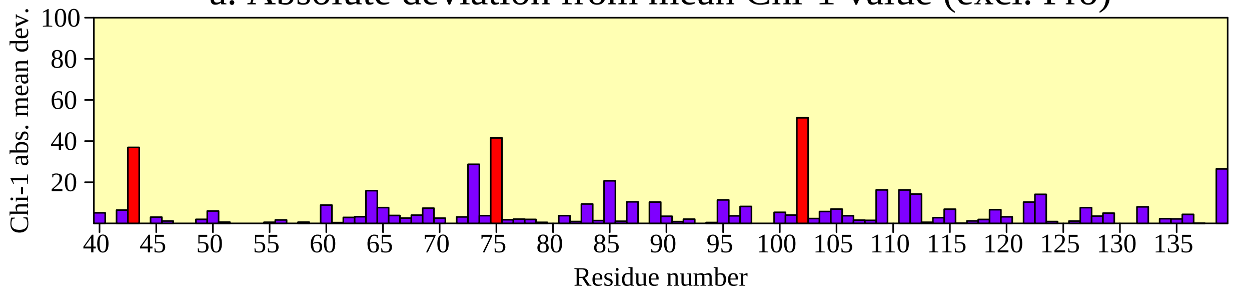

b. Absolute deviation from mean of omega torsion

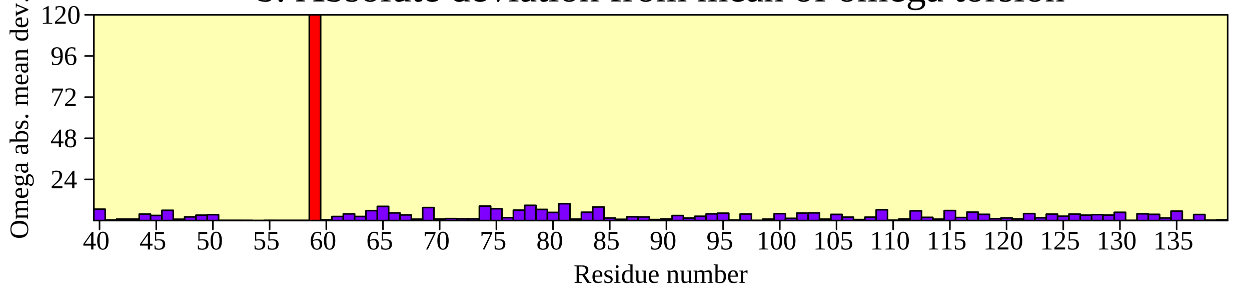

c. C-alpha chirality: abs. deviation of zeta torsion

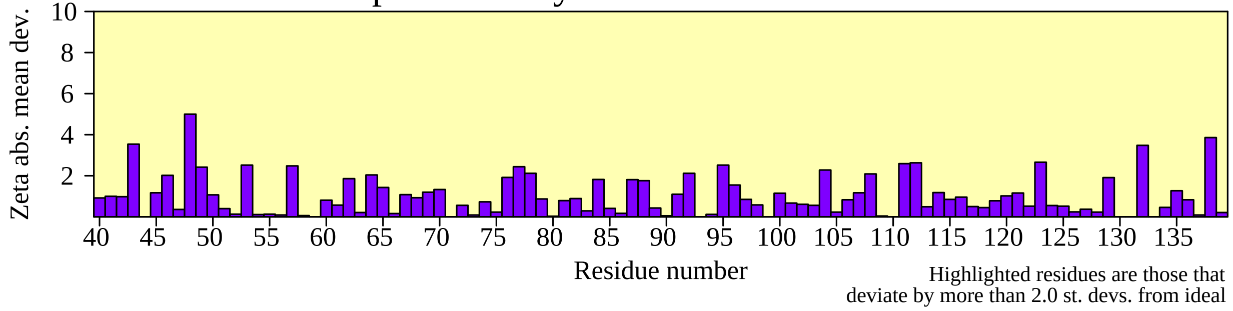

Highlighted residues are those that deviate by more than 2.0 st. devs. from ideal

d. Secondary structure & estimated accessibility

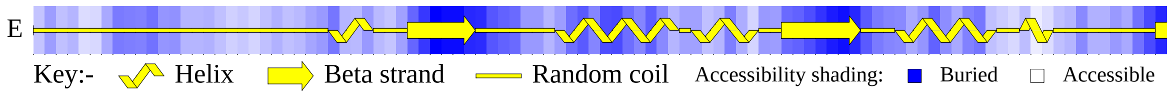

e. Sequence & Ramachandran regions

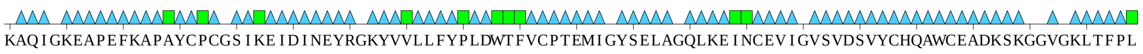

f. Max. deviation (see listing)

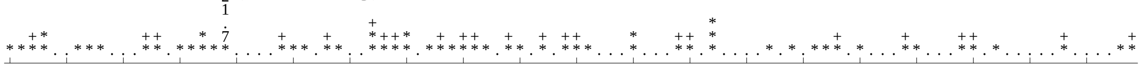

g. G-factors

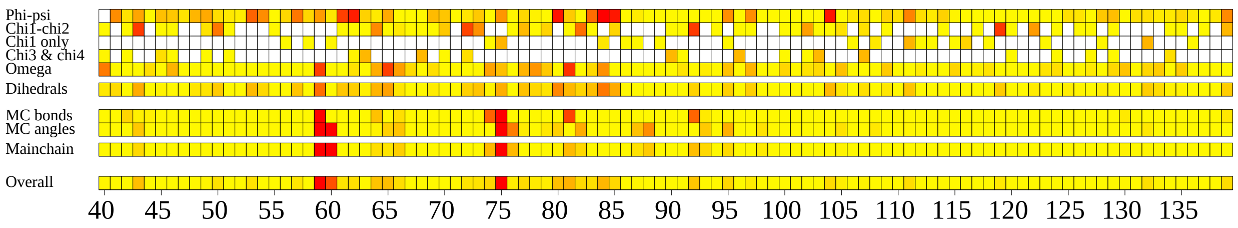

Residue properties

Model_12

a. Absolute deviation from mean Chi-1 value (excl. Pro)

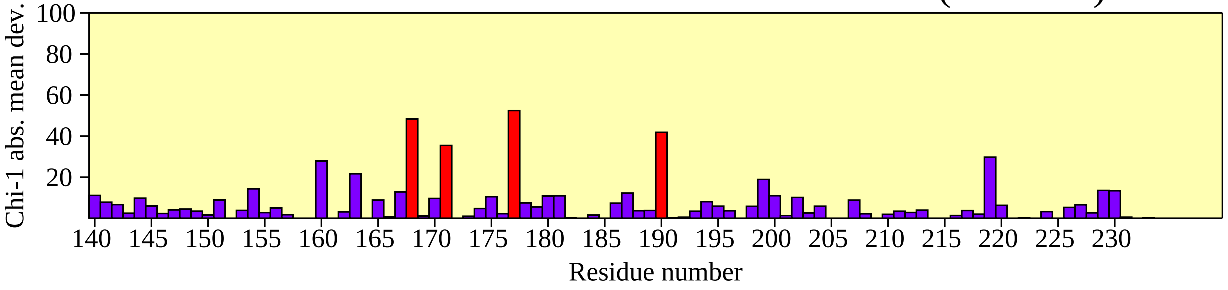

b. Absolute deviation from mean of omega torsion

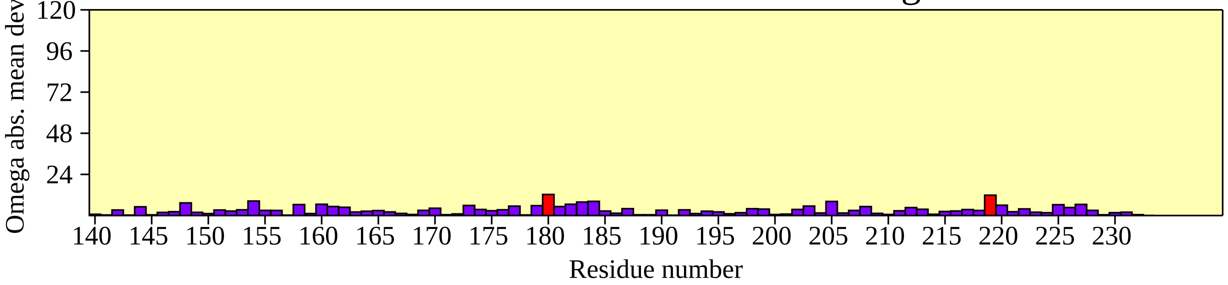

c. C-alpha chirality: abs. deviation of zeta torsion

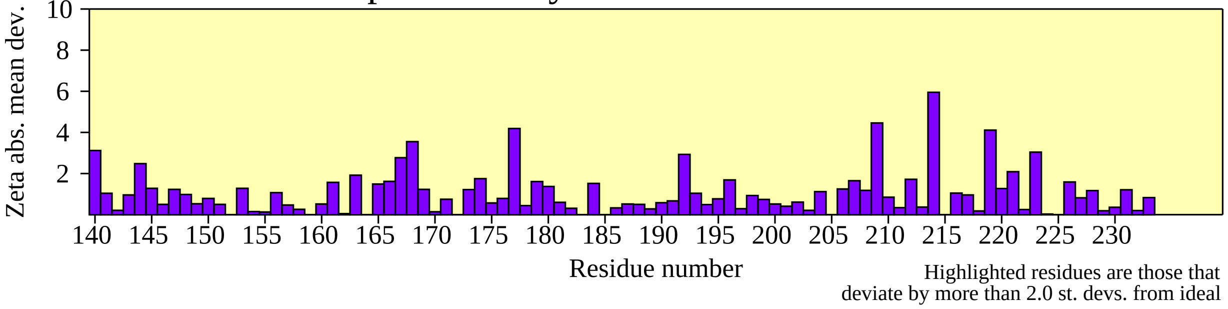

Highlighted residues are those that deviate by more than 2.0 st. devs. from ideal

d. Secondary structure & estimated accessibility

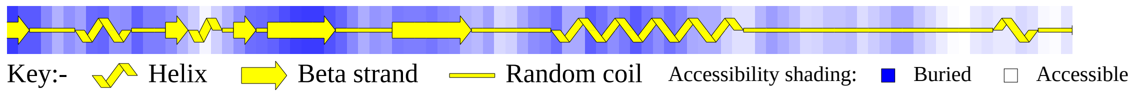

e. Sequence & Ramachandran regions

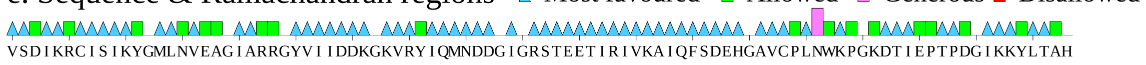

f. Max. deviation (see listing)

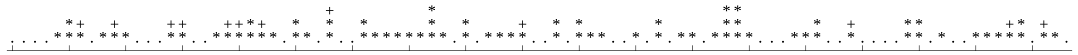

g. G-factors

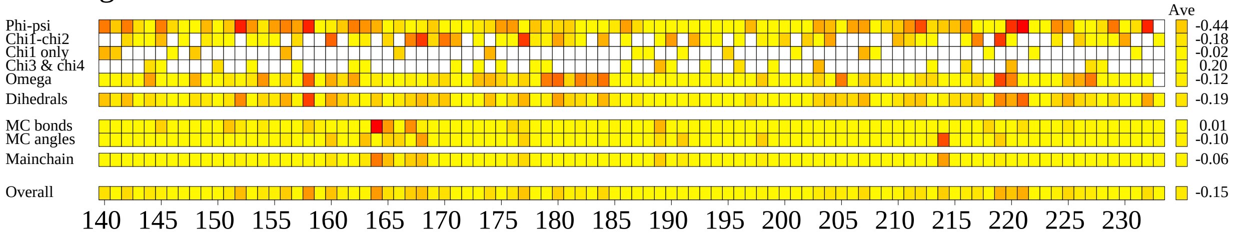

RMS distances from planarity

23_128_72

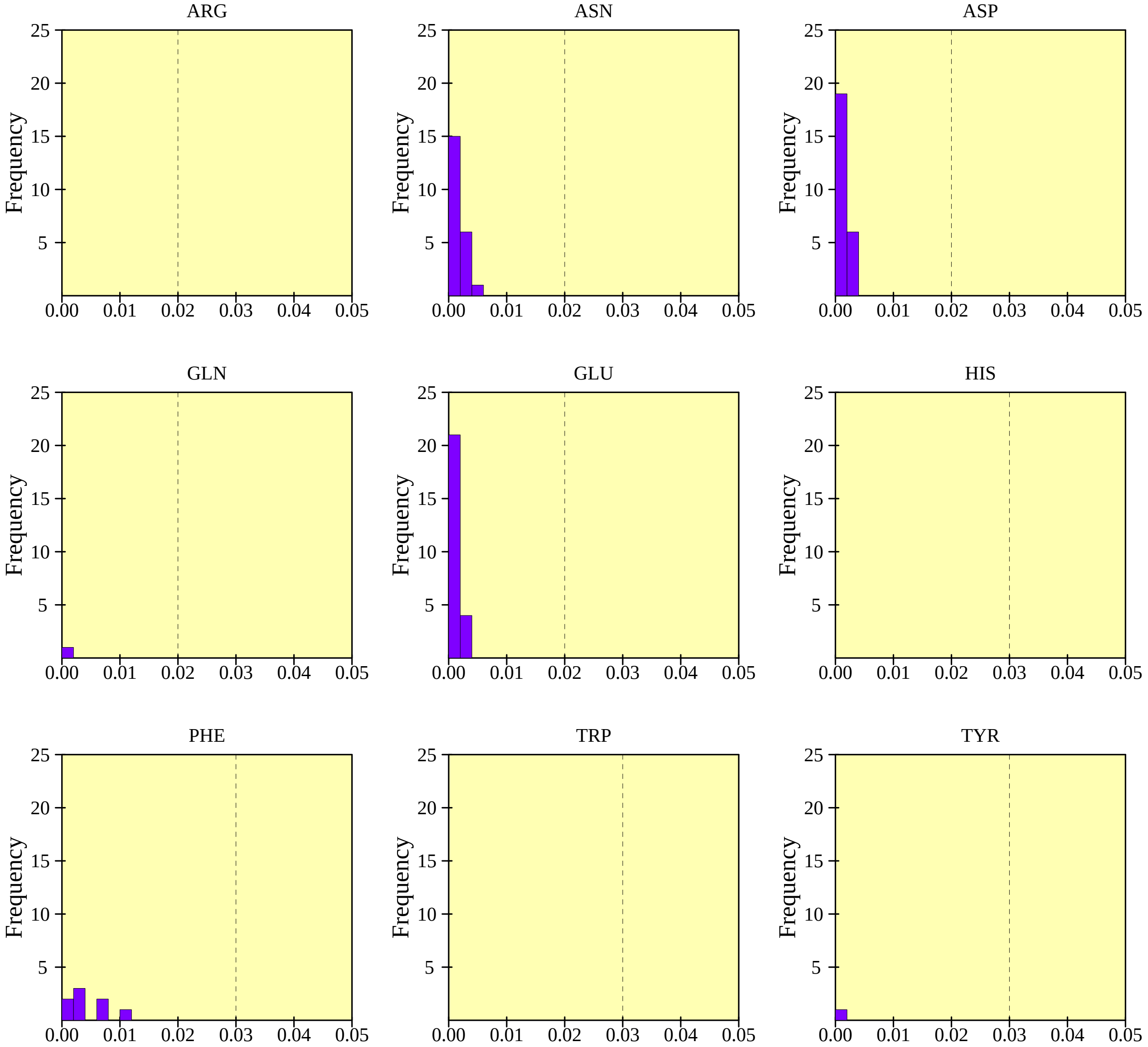

Histograms showing RMS distances of planar atoms from best-fit plane.
Black bars indicate large deviations from planarity: RMS dist > 0.03 for rings, and > 0.02 otherwise.

RMS distances from planarity

Model_12

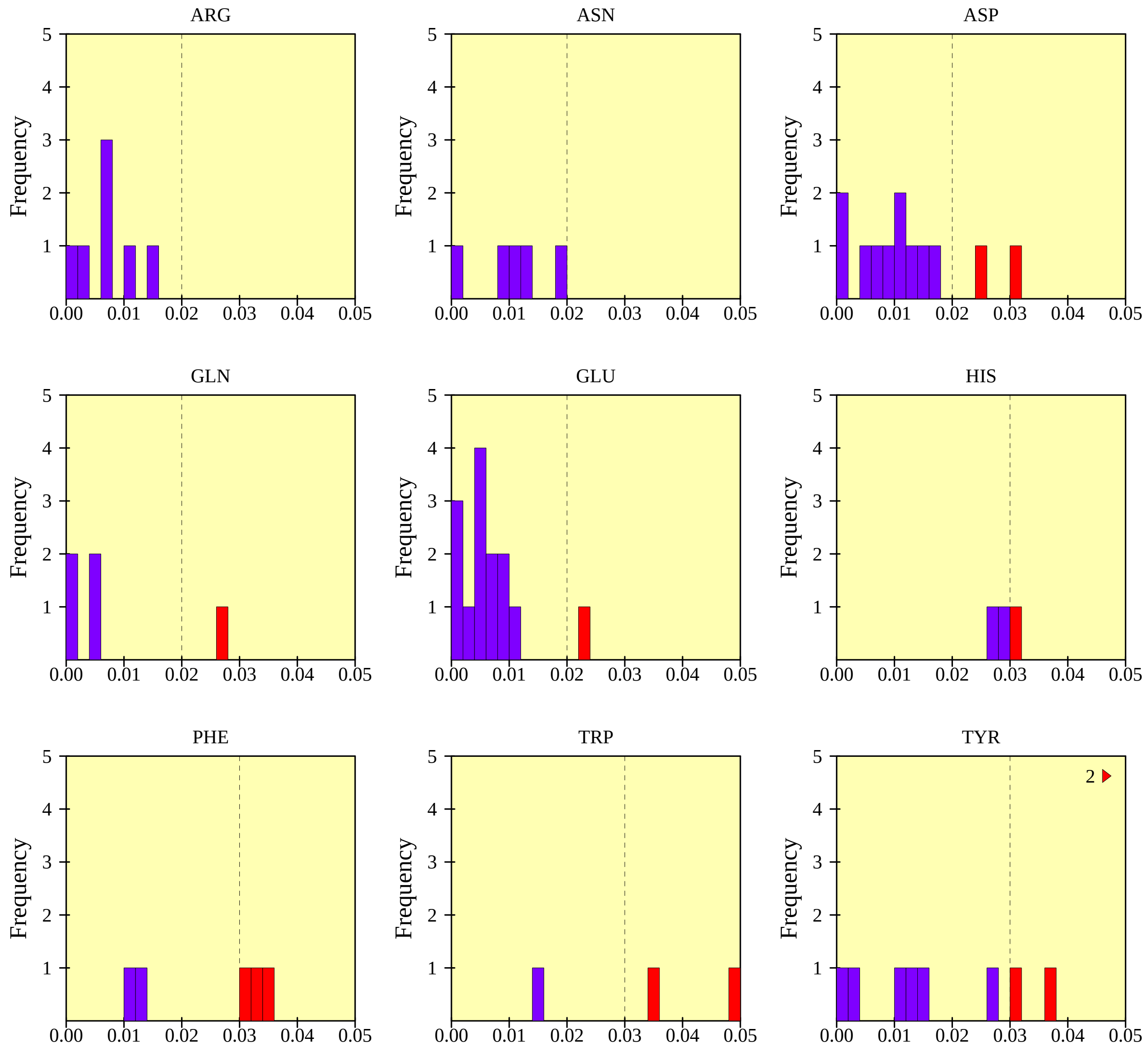

Histograms showing RMS distances of planar atoms from best-fit plane.
Black bars indicate large deviations from planarity: RMS dist > 0.03 for rings, and > 0.02 otherwise.

▶ signifies data points off the graph in the direction shown.
