# Supplementary figures and images for: In-silico prediction and modeling of the Entamoeba histolytica proteins: Serine-rich Entamoeba histolytica protein and 29 kDa Cysteine-rich protease
Source: PeerJ. 2017 Jun 28;5:e3160. doi: 10.7717/peerj.3160 (PMC5493030; doi:10.7717/peerj.3160)

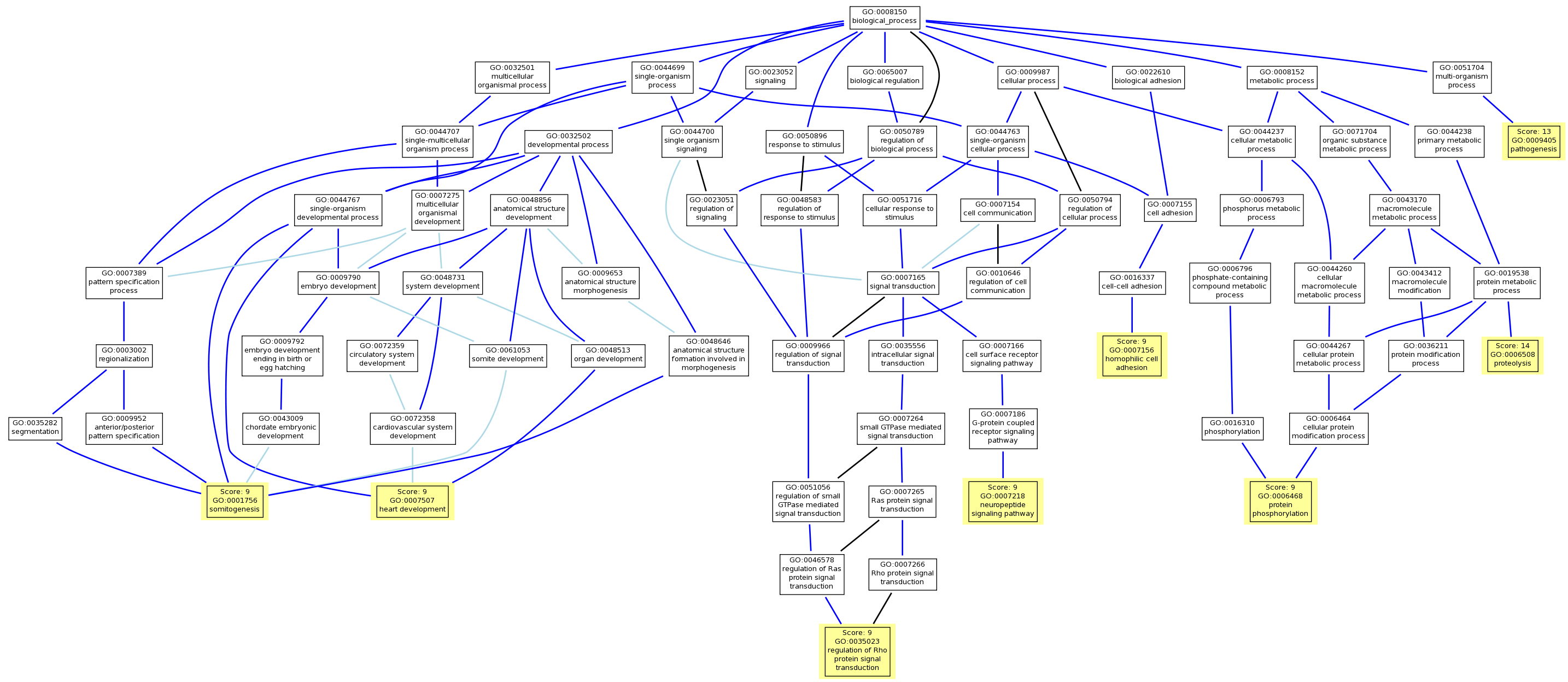

Supplement: Supplemental Information 8 — The functional ontology of SREHP shows that it is involved in pathogenesis, protein phosphrylation and in regulation of signal transduction. [file peerj-05-3160-s008.png]

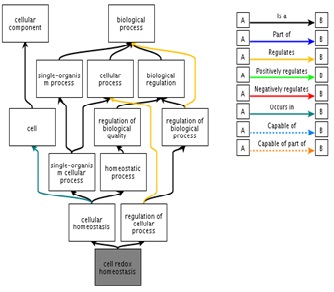

Supplement: Supplemental Information 9 — Functional ontology of Eh29 protein shows that it is involved in the mechanism of cell redox homeostasis. [file peerj-05-3160-s009.jpg]
