# Supplementary material for: In-silico prediction and modeling of the Entamoeba histolytica proteins: Serine-rich Entamoeba histolytica protein and 29 kDa Cysteine-rich protease
Source: PeerJ. 2017 Jun 28;5:e3160. doi: 10.7717/peerj.3160 (PMC5493030; doi:10.7717/peerj.3160)
Supplement: Supplemental Information 10 — The table summarizes the 51 antigenic regions found in the Gal/GalNAc lectin. The sequence details with start and end position has been tabulated. [file peerj-05-3160-s010.docx]

| **Sr. No.** | **Start Position** | **Sequence** | **End Position** |
| --- | --- | --- | --- |
| 1 | 4 | LLLNILLLCCLA | 15 |
| 2 | 23 | ADIDYYDL | 30 |
| 3 | 41 | SWYHSYTHQYDVFYYLA | 57 |
| 4 | 61 | WRHFVWT | 67 |
| 5 | 102 | KQDFCQKEYAYPIEK | 116 |
| 6 | 120 | DWDNVPVDE | 128 |
| 7 | 140 | TCFKYAAKRPLAYVYL | 155 |
| 8 | 166 | EAYDVCRM | 173 |
| 9 | 202 | TSKCIID | 208 |
| 10 | 215 | NTHLAIILG | 223 |
| 11 | 225 | TDSTVIKSLQEKLSVLSQLTTVDGVTIYYLKG | 256 |
| 12 | 270 | KYETLVKY | 277 |
| 13 | 280 | GQGQVDPLVN | 289 |
| 14 | 307 | GTMVVLM | 313 |
| 15 | 331 | NISVHTVVL | 339 |
| 16 | 346 | KITYSALKLVSLGPHYH | 362 |
| 17 | 402 | NRCTCPMCCENDCFYTSCDVETGSCIPWP | 430 |
| 18 | 436 | AKKECPATCVGLYECKDLEGCVVTKYNASCEPKVKCMVPYC | 476 |
| 19 | 482 | LKEVCKQ | 488 |
| 20 | 500 | SDGYCWSYTC | 509 |
| 21 | 520 | KHGNLCT | 526 |
| 22 | 531 | NCQEYVCDS | 539 |
| 23 | 541 | QRCTVQEKVCVKTS | 554 |
| 24 | 556 | YIEMSCYVAKC | 566 |
| 25 | 575 | NRLSCDTYSSC | 585 |
| 26 | 590 | TGSVCKCD | 597 |
| 27 | 602 | NQCKCNK | 608 |
| 28 | 619 | KHEICDY | 625 |
| 29 | 629 | KPKCIVSECTE | 639 |
| 30 | 642 | VRDGCLIK | 649 |
| 31 | 661 | ENVDCSN | 667 |
| 32 | 680 | TMCKPYYSATCLNGQCVVQAVGDVSNVGCGYCS | 712 |
| 33 | 717 | NVITYHDD | 724 |
| 34 | 745 | NSYSCVF | 751 |
| 35 | 764 | ICAECSSLTCPA | 775 |
| 36 | 789 | GTCKATVKPTPSCSVCEK | 806 |
| 37 | 818 | LERKVTL | 824 |
| 38 | 834 | IPKDCVNEQCIPRTYVDCLA | 853 |
| 39 | 860 | EIYKFYLPCQAYVTATYHYSSLFNLTSYKLHLPQ | 893 |
| 40 | 903 | KEAYCTY | 909 |
| 41 | 915 | ECKTCSL | 921 |
| 42 | 929 | EEIDLCA | 935 |
| 43 | 956 | PNFDCQPIECKIQEIVI | 972 |
| 44 | 1013 | EQVECASTVCQN | 1024 |
| 45 | 1026 | NSCPIIADVEK | 1036 |
| 46 | 1056 | GTTYLCKFVQL | 1066 |
| 47 | 1085 | LNNACLKYKCVE | 1096 |
| 48 | 1140 | TKKTCTVSE | 1148 |
| 49 | 1157 | QGRCFYCQCSYLDGSSVLT | 1175 |
| 50 | 1183 | EYYDLDACGN | 1192 |
| 51 | 1219 | NVGAIAAATTVAVVVVAVVVALIVVSIGLFKTYQLVSS | 1256 |
